# Ivy Optimization Algorithm Combining Sine–Cosine Operator and Adaptive T-Distribution and Its Engineering Application

**DOI:** 10.3390/biomimetics11070468

**Published:** 2026-07-03

**Authors:** Zhenkun Lu, Jianyong Zhu, Dingfeng Lu, Hongze Lv, Haolin Gan, Zicong An

**Affiliations:** 1 School of Robot Engineering, Wenzhou University of Technology, Wenzhou 325035, China; 2 Vocational Training Department, Rizhao Technician College, Rizhao 276800, China; 3 School of Renewable Energy, Inner Mongolia University of Technology, Ordos 017010, China

**Keywords:** Ivy Optimization Algorithm, improved Logistics chaotic mapping, sine–cosine operator, adaptive t-distribution mutation, engineering optimization design

## Abstract

The Ivy Optimization Algorithm (IVY) is a novel swarm intelligence optimization algorithm that simulates the phototropic growth mechanism of plants. To comprehensively improve the overall optimization performance, this paper proposes an enhanced Ivy Optimization Algorithm (LSIVY) integrating improved Logistics chaotic mapping, sine–cosine operator, and adaptive t-distribution mutation strategy. Firstly, an improved cascaded Logistics chaotic mapping is used for population initialization. The double arcsine transformation improves the ergodicity and uniformity of chaotic sequences, so that initial solutions are distributed more evenly in the search space, population diversity is enhanced, and premature convergence is suppressed. Secondly, the sine–cosine operator is embedded into the position update mechanisms of IVY growth, climbing, and propagation evolution. Nonlinearly decreasing control parameters realize adaptive switching between global exploration and local exploitation and accelerate convergence. Thirdly, an adaptive t-distribution mutation strategy is designed to dynamically adjust mutation intensity according to the iteration cycle and implement directional perturbation at the optimal solution position. It combines the large-scale exploration advantage of the Cauchy distribution and the local fine search merit of the Gaussian distribution, which significantly improves the ability to escape from local optima. Comparative experiments with eight mainstream metaheuristics (DE, WOA, GWO, HHO, DBO, MBWO, AOO, native IVY) are conducted with 30 independent runs on 30-dimensional CEC 2014 (30 test functions) and CEC 2020 (10 composite functions). Quantitatively, LSIVY achieves 20~30 orders of magnitude higher optimization accuracy than standard IVY on unimodal functions, and its average standard deviation across all benchmarks drops by 4–6 orders of magnitude. LSIVY ranks first on all CEC 2020 composite functions, reducing over 30% of iterations compared with native IVY. Three classical constrained mechanical design problems (three-bar truss, cantilever beam, pressure vessel) are adopted for engineering verification. In the pressure vessel case, the average manufacturing cost of LSIVY is reduced by 9.2% against standard IVY, and the standard deviation of three engineering cases decreases by 2–3 orders on average, demonstrating remarkable robustness. The proposed algorithm not only improves the theoretical system of plant-inspired swarm intelligence algorithms but also has great application prospects in mechanical structure lightweight design, industrial equipment cost optimization, and other practical engineering fields.

## 1. Introduction

A broad spectrum of practical optimization tasks emerging in scientific computation, engineering design, production scheduling, and intelligent control can be abstracted into complex optimization formulations characterized by nonlinearity, non-convexity, multiple constraints, and numerous local extrema [1]. Conventional gradient-based mathematical programming approaches impose stringent prerequisites on objective functions and fail to guarantee globally optimal solutions, or even produce feasible search trajectories, when tackling high-dimensional, non-differentiable, non-convex optimization problems contaminated by heavy noise [2,3]. Driven by rapid advances in modern industrial and information technologies, engineering design, intelligent manufacturing, data analytics, resource allocation, and network optimization have spawned a massive class of intricate optimization problems marked by high dimensionality, strong nonlinearity, multiple modal landscapes, and rigid constraint boundaries. Such problems exhibit exponentially expanding solution spaces, which render traditional gradient-driven deterministic algorithms and early heuristic search strategies ineffective [4]. These classical methods are prone to stagnating at local optima, suffer sluggish convergence, or even become computationally intractable due to combinatorial explosion, thereby failing to satisfy the solution demands of large-scale, strongly coupled industrial systems [5,6]. Against this backdrop, the self-organized collaborative behaviors and adaptive evolutionary characteristics of social swarms in nature (e.g., ant colonies, bee colonies, bird flocks, and fish schools) offer a novel paradigm for addressing the above bottlenecks. Each swarm agent executes simple decision rules without centralized command and control [7]. Through local information interaction, environmental perception, and historical experience sharing, the whole population exhibits emergent collective intelligence to accomplish complex survival tasks, including foraging, long-distance migration, and nest construction [8]. The core underlying logic—simple decentralized individuals cooperate to achieve sophisticated global objectives—matches the solution requirements of complex multi-extremum optimization problems perfectly [9].

Swarm intelligence optimization algorithms construct distributed and iterative random search models by simulating biological group behaviors, physical motion laws, or plant growth characteristics [10,11]. They do not rely on gradient information, have strong robustness and outstanding global search ability, and are easy to implement. They have become the mainstream technical method for solving complex optimization problems [12]. From the perspective of disciplinary development, the evolution of swarm intelligence optimization algorithms follows the closed-loop logic of “biological inspiration–modeling abstraction–algorithm implementation–application verification–mechanism optimization”. It is essentially the digital reproduction and engineering adaptation of the evolution law of swarm intelligence in nature [13]. Compared with traditional optimization algorithms, swarm intelligence algorithms have unique advantages such as gradient-free dependence, strong robustness, easy parallel implementation, and adaptability to multiple types of problems. They can effectively deal with many unstructured and complex optimization scenarios without analytical solutions in the real world [14,15]. At present, with the new generation artificial intelligence development plan listing swarm intelligence as a key direction of artificial intelligence theory, the research of swarm intelligence optimization algorithms has shifted from single-algorithm design to multi-algorithm coordination, cross-field integration, and in-depth coupling of theory and application [16,17]. The core goal is not only to build a better optimization framework, but also to explore the internal mechanism of swarm intelligence emergence and provide solid theoretical support and technical guarantee for collaborative decision-making and intelligent optimization of complex systems. It has important academic research value and broad engineering application prospects [18,19].

In recent years, various swarm intelligence optimization algorithms have emerged, such as Particle Swarm Optimization (PSO) [20], Differential Evolution (DE) [21], Gray Wolf Optimizer (GWO) [22], Whale Optimization Algorithm (WOA) [23], Harris Hawks Optimization (HHO) [24], Dung Beetle Optimizer (DBO) [25], Multiple Objective Beluga Whale Optimization (MBWO) [26], Animated Oat Optimization algorithm (AOO) [27], etc. These algorithms have been widely used in function optimization, path planning, structural engineering design, power system scheduling, machine learning parameter optimization, image processing, and other fields, showing strong practicality and scalability. With the expansion of optimization scale and increasingly complex constraints, higher requirements are put forward for the convergence speed, optimization accuracy, stability, and local optimum escaping ability of algorithms [28]. It is of great theoretical value and engineering significance to design improved swarm intelligence algorithms with better performance and wider applicability [29]. The Ivy Optimization Algorithm (IVY) is a novel plant growth-inspired intelligent optimization algorithm proposed by Ghasemi et al. in 2024 [30]. It simulates the life cycle behaviors of IVY in a natural environment, such as creeping growth, climbing support, phototropic propagation, and survival of the fittest [31]. It achieves iterative optimization through growth rate control, strong-neighbor guidance, global optimal guidance, and a survivor screening mechanism. Compared with traditional swarm intelligence algorithms, IVY has the characteristics of few parameters, a clear structure, strong local exploitation ability, and stable late convergence. It shows good potential in continuous numerical optimization problems [32]. However, with the increase in application scenario complexity, the inherent defects of standard IVY are gradually exposed. (1) Population initialization relies on random distribution, resulting in poor ergodicity and insufficient diversity of the initial solution space, which easily makes the algorithm converge prematurely to local optima. (2) Fixed growth rate and position update mechanism make it difficult to dynamically balance global exploration and local exploitation, and search efficiency decreases when facing multimodal and high-dimensional problems. (3) Severe population homogeneity in the late iteration lacks an effective mutation disturbance strategy, making it difficult to jump out of local optima. (4) Insufficient stability when facing highly constrained engineering problems, and optimization accuracy and convergence speed need to be improved.

In recent years, some scholars have improved the IVY algorithm. For example, the IVY is combined with a Gaussian guidance mechanism for unmanned vehicle path planning, and the growth rate is improved through a spiral decay strategy; opposition-based learning, chaotic mapping, and adaptive weight strategies are adopted to improve algorithm diversity and search balance ability. Most existing improved IVY variants only adopt a single optimization strategy, without an integrated collaborative improvement framework covering population initialization, iterative position update, and late-escape mutation. This is the core motivation of this paper to select IVY as the base algorithm. The standard IVY has an exclusive local climbing exploitation module by nature, with a concise structure and few hyperparameters, making it a promising basic model in plant-based optimizers. Nevertheless, its inherent defects, including random initialization, fixed update rules, and a lack of adaptive disturbance, restrict its performance on high-dimensional and strongly constrained engineering problems. Therefore, we design a three-layer hybrid improved strategy named LSIVY to make up for the deficiencies. Relevant theoretical logic has been added to the introduction to strengthen the rationality of topic selection. Moreover, this improved mapping is rarely applied systematically to the plant-inspired IVY algorithm, which is a basic improvement of this work. The existing studies mostly integrate it into PSO, GWO, and other algorithms, and a few researchers adapt it to the unique growth, climbing, and propagation update mechanism of IVY. The adaptive integration of the sine–cosine operator and the native position update logic of IVY is another innovation of this paper. Most existing mutation strategies are fixed-intensity Gaussian mutation and Cauchy mutation with fixed characteristics, which cannot meet the search requirements in the whole iteration process: Cauchy mutation is suitable for large-scale global jumps, while Gaussian mutation is for local fine search, and a single mutation cannot switch modes dynamically. A few studies have tried to introduce t-distribution mutation, but they mostly adopt fixed degrees of freedom, without realizing adaptive adjustment combined with iteration and directional perturbation on the optimal individual.

To overcome the shortcomings of standard IVY, this paper proposes an Ivy Optimization Algorithm (LSIVY) integrating improved Logistics chaotic mapping, sine–cosine operator, and adaptive t-distribution mutation. The main innovations are as follows:

(1) Improved cascaded Logistics chaotic mapping is adopted for population initialization to improve the uniformity and diversity of initial solutions.

(2) Sine–cosine operator is embedded into position update formulas to dynamically balance global exploration and local exploitation.

(3) An adaptive t-distribution mutation strategy is proposed to adaptively adjust disturbance intensity according to iteration cycle and enhance the ability to escape from local optima.

(4) The superiority of the algorithm is verified on CEC 2014, CEC 2020 function suites, and three types of engineering optimization problems.

## 2. Standard Ivy Optimization Algorithm

The Ivy Optimization Algorithm (IVY) is a novel plant-inspired metaheuristic that mimics the natural growth, climbing, and propagation characteristics of ivy plants. Within the D-dimensional search space, each feasible solution is abstracted as an IVY individual, and a random uniform sampling strategy is utilized to generate the initial population [33]. By simulating the phototropic growth, sunlight-oriented climbing, and population propagation cycles of ivy, the algorithm iteratively evolves high-quality candidate solutions, where a survivor screening rule is implemented to filter elite individuals for the subsequent generation [34,35]. Centered on the static growth and dynamic climbing logic of ivy, IVY features a streamlined structural framework, a small set of hyperparameters, and interpretable iterative updating rules. Equipped with a dedicated local exploitation module, it exhibits superior adaptability to continuous numerical optimization tasks compared with conventional metaheuristics. In accordance with the complete life cycle of ivy, the evolutionary workflow of IVY can be decomposed into five core functional modules: population initialization, autonomous growth update, neighbor-guided climbing, population propagation evolution, and elite survivor selection [36]. Set the internal population size of the algorithm as $N_{pop}$, the dimension of the optimization problem as D. The multi-dimensional position vector corresponding to the i-th ivy individual in the search space can be defined as Equation (1):

(1) $$\begin{array}{c} X_{i}=[x_{i,1},x_{i,2},\ldots ,x_{i,D}],i=1,2,\ldots ,N_{pop} \end{array}$$

**(1) Population initialization**

The standard IVY algorithm constructs the initial population by uniform random generation. The initial positions of individuals are restricted by upper and lower boundaries to cover the complete search space. The specific calculation formula is as follows [37]:

(2) $$\begin{array}{c} X_{i}=LB+rand(1,D)⊙(UB-LB) \end{array}$$

where LB and UB represent the lower and upper bounds of the optimization problem solution space, respectively. rand(1,D) is a *D*-dimensional vector randomly generated in the interval [0,1]. ⊙ is the Hadamard product operator for point-to-point multiplication between vectors.

**(2) Growth rate update**

Growth rate is the core parameter of IVY, which directly determines the maximum moving step of IVY individuals in a single iteration and affects the exploration and exploitation ability of the algorithm. The initial growth rate is assigned based on the initial population position and dynamically updated in the iteration stage. The initial assignment formula and iterative update formula are shown in Equation (3) and Equation (4), respectively [38]:

(3) $$\begin{array}{c} GV_{i}=X_{i}⊘(X_{max}-X_{min}) \end{array}$$

(4) $$\begin{array}{c} GV_{i}(t+1)=rand^{2}⊙(N^{D}(0,1)⊙GV_{i}(t)) \end{array}$$

where ⊘ is the Hadamard division operator. $X_{max}$ and $X_{min}$ are the population position extremes. $N^{D}(0,1)$ represents a *D*-dimensional random vector obeying the standard normal distribution. $rand^{2}$ is the square value of a random number in [0,1].

**(3) Roaming and climbing mechanism**

The roaming and climbing mechanism is the key to local exploitation of IVY. The algorithm screens high-quality individuals with strong neighbors in the population according to the fitness value and guides ordinary ivy individuals to climb toward high-quality individuals [39]. The strong neighbor screening rule and climbing position update formula are as follows:

(5) $$I_{ii}=\left\{\begin{array}{ll} I_{j-1}^{s}, & I_{i}=I_{j}^{s}\\ I_{i}, & I_{i}=I_{best} \end{array}\right.\;$$

(6) $$\begin{array}{c} X_{i}^{new}=X_{i}+|N^{D}(0,1)|⊙(I_{ii}-X_{i})+N^{D}(0,1)⊙GV_{i} \end{array}$$

where $I_{ii}$ is the optimal climbing neighbor corresponding to the individual. $I_{best}$ is the global optimal IVY individual position in the current iteration cycle.

**(4) Propagation and evolution mechanism**

To make up for the insufficient global search ability of the climbing mechanism, IVY adds a propagation and evolution mechanism. Guided by the global optimal individual, all IVY individuals are driven to gather toward the optimal solution area to complete a large-scale global search [40]. The corresponding position update formula is

(7) $$\begin{array}{c} X_{i}^{new}=X_{best}⊙(rand+N^{D}(0,1)⊙GV_{i}) \end{array}$$

**(5) Survivor selection mechanism**

To ensure the constant population size and steady improvement of overall quality, the algorithm introduces a survivor selection mechanism. After each iteration, the parent original population and offspring new population are merged, sorted in ascending order according to the fitness value, and the top $N_{pop}$ high-quality individuals are selected as the next-generation iterative population. This mechanism can effectively eliminate inferior individuals and stabilize the optimization performance of the population. The update formula is as follows:

(8) $$\begin{array}{c} X=[X_{1}^{M/S},X_{2}^{M/S},\ldots ,X_{N_{pop}}^{M/S}] \end{array}$$

## 3. Ivy Optimization Algorithm Combining Sine–Cosine Operator and Adaptive T-Distribution (LSIVY)

### 3.1. Improved Logistics Chaotic Mapping for Population Initialization

The standard Logistics chaotic mapping has the problem of a dense middle area and sparse both ends. In this paper, the double-arcsine cascaded improved Logistics mapping (ILM) is adopted to significantly improve the uniformity and ergodicity of chaotic sequences, so as to optimize the effect of population initialization [41]. The corresponding chaotic iterative calculation formula is

(9) $$\left\{\begin{array}{l} x_{n+1}^{\prime }=4x_{n^{\prime }}(1-x_{n^{\prime }})\\ y_{n}^{\prime }=\frac{1}{\pi }\arcsin(2x_{n+1}^{\prime }-1)-\frac{1}{2}\\ x_{n+1}=4y_{n^{\prime }}(1-y_{n}^{\prime })\\ y_{n}=\frac{1}{\pi }\arcsin(2x_{n+1}-1)-\frac{1}{2} \end{array}\right.$$

The generated uniform chaotic sequence is linearly mapped to the optimization problem solution space to complete the initialization of the IVY population. The calculation formula is as follows [42]:

(10) $$\begin{array}{c} Z_{i}=LB_{i}+(UB_{i}-LB_{i})\cdot y_{n} \end{array}$$

Compared with random initialization and traditional Logistics chaotic initialization, the initial population generated by this improved strategy has stronger ergodicity, can fully cover the complete solution space, and avoids local aggregation of individuals. It improves the quality of the initial population from the iteration source and effectively suppresses the premature convergence of the algorithm.

### 3.2. Integration of Sine–Cosine Operator Strategy

The sine–cosine algorithm (SCA) realizes bidirectional search of individuals moving away from or close to the optimal solution area based on the periodic fluctuation characteristics of sine and cosine trigonometric functions, which can dynamically balance the global exploration and local exploitation capabilities of the algorithm [43,44]. In this paper, the operator is embedded into the two core update stages of IVY: climbing and propagation, to optimize the original single position update mode. The improved position update formula is as follows:

(11) $$X_{i}^{t+1}=\left\{\begin{array}{ll} r_{1}\sin r_{2}\times |X_{best}^{t}-X_{i}^{t}|+X_{best}^{t}, & r_{3}>0.5\\ r_{1}\cos r_{2}\times |X_{best}^{t}-X_{i}^{t}|+X_{best}^{t}, & r_{3}\leq 0.5 \end{array}\right.\;$$

where the control factor $r_{1}$ adopts a nonlinear decreasing mode, the calculation formula is [45]:

(12) $$\begin{array}{c} r_{1}=a-t\cdot \frac{a}{T_{max}} \end{array}$$

where the constant a=2, t is the current number of iterations. $T_{max}$ is the maximum number of iterations. $r_{2}\in [0,2\pi ]$ is used to adjust the moving distance between the individual and the optimal solution. $r_{3}\in [0,1]$ is a random decision coefficient to switch the sine and cosine update modes with equal probability [46]. A large $r_{1}$ value in the early iteration strengthens the global exploration ability, while a small $r_{1}$ in the late iteration focuses on local fine exploitation, comprehensively optimizing the convergence speed and optimization accuracy of the algorithm.

### 3.3. Adaptive T-Distribution Mutation Strategy

The *t*-distribution is a continuous probability distribution model with adjustable degrees of freedom. Its distribution characteristics can be dynamically switched: when the degree of freedom is small, it approaches the Cauchy distribution with large-scale global disturbance ability [47]. When the degree of freedom is large, it approaches the Gaussian distribution, suitable for local fine search. To solve the problems of population homogeneity and easy falling into local optima in the late iteration of standard IVY, this paper designs an adaptive *t*-distribution mutation strategy with iteration times as the degree of freedom to conduct directional disturbance on the global optimal individual [48]. The mutation update formula is as follows:

(13) $$\begin{array}{c} X_{new}^{t}=X_{best}^{t}+\omega \cdot TD(t)\cdot X_{best}^{t} \end{array}$$

where the adaptive weight coefficient ω decreases linearly with the iteration process, taking into account the early disturbance exploration and late elite retention requirements. The calculation formula is [49]

(14) $$\begin{array}{c} \omega =a+(b-a)\cdot \frac{T_{max}-t}{T_{max}} \end{array}$$

Set the acceptance probability threshold to randomly determine whether to update the global optimal solution. The update rule is

(15) $$X_{best}=\left\{\begin{array}{ll} X_{best}, & pe>0.5\\ X_{new}, & pe\leq 0.5 \end{array}\right.\;$$

This strategy integrates the dual advantages of the Cauchy distribution and the Gaussian distribution and adaptively adjusts the disturbance intensity. It can not only help the algorithm jump out of the local optimal trap, but also avoid excessive disturbance destroying high-quality elite solutions, greatly improving the algorithm’s robustness under complex working conditions.

### 3.4. Algorithm Implementation Steps and Pseudocode

The pseudocode of the IVY Optimization Algorithm combining sine–cosine operator and adaptive t-distribution (LSIVY) is as follows Algorithm 1:
**Algorithm 1:** Ivy Optimization Algorithm Combining Sine–Cosine Operator and Adaptive T-Distribution (LSIVY)**Input**: Population size $N_{pop}$, maximum iterations $T_{max}$, solution dimension D, search space boundaries LB,UB. **Output**: Global optimal position $X_{best}$, optimal fitness value $f_{best}$.Step 1. Initialize basic algorithm parameters, set fixed parameters a=2, b=1, mutation acceptance probability pe=0.5;Step 2. Generate uniformly distributed initial ivy population based on improved Logistics chaotic mapping Equations (9) and (10);Step 3. Calculate the fitness values corresponding to all initial individuals, and screen the initial global optimal individual $X_{best}$, and optimal fitness $f_{best}$;Step 4. Assign the initial population growth rate $GV_{i}$ according to Equation (3);Step 5. While $t\leq T_{max}$   5.1. Iteratively update the ivy individual growth rate according to Equation (4);    5.2. Execute the roaming and climbing mechanism, and introduce the sine–cosine operator Equation (11) to update individual positions;    5.3. Start the propagation and evolution mechanism to complete the secondary position update of the population;    5.4. Call the adaptive t-distribution mutation strategy to perturb the optimal individual according to Equations (13)–(15);    5.5. Perform boundary constraint processing on out-of-bounds individuals and reset their positions to the solution space;    5.6. Recalculate the fitness values of all offspring individuals;    5.7. Execute the survivor screening mechanism and update the global optimal individual and optimal fitness;    5.8. Iteration counter auto-increment: t=t+1;Step 6. End WhileStep 7. Return *X_best_*, *f_best_*

The complexity analysis of the algorithm is as follows. To verify that the multi-strategy improvement scheme does not add additional computing burden to the algorithm, this paper decomposes and analyzes the time complexity of LSIVY from three dimensions: the initialization module of LSIVY adopts improved cascaded Logistics chaotic mapping instead of random sampling. The process of generating chaotic sequences only carries out basic trigonometric transformation and linear mapping operations for each individual, and no high-dimensional matrix inversion or complex nonlinear solution is involved. For a population with size $N_{pop}$ and optimization dimension *D*, the time consumption of chaotic sequence generation and individual position assignment is $O(N_{pop}D)$. Afterwards, the algorithm traverses all individuals to calculate the initial fitness value, and the time complexity of fitness evaluation is $O(N_{pop})$. Thus, the total time complexity of the initialization stage is $O(N_{pop}D+N_{pop})$. Compared with the random initialization of standard IVY, the chaotic part only adds a small number of elementary arithmetic operations, and there is no promotion of the asymptotic complexity order. A complete iteration of LSIVY includes five core links: growth rate update, climbing position update embedded with sine–cosine operator, propagation evolution, adaptive t-distribution mutation, and survivor screening sorting. (1) Growth rate update: Each individual executes vector Hadamard operation, with complexity $O(N_{pop}D)$. (2) Sine–cosine embedded climbing and propagation update: Two rounds of individual position transformation, trigonometric function and linear scaling are all lightweight calculations, and the total complexity is $O(N_{pop}D)$. (3) Adaptive t-distribution directional mutation: Only the global optimal individual is subjected to probability distribution perturbation, without traversing the whole population, and the computational cost of this link is far lower than full-population mutation; the time complexity is only O(D). (4) Boundary constraint correction: Traverse all individuals to judge and clamp the out-of-bounds dimensions, complexity $O(N_{pop}D)$. (5) Survivor selection sorting: Merge parent and offspring populations, sort all individuals by fitness in ascending order, and the time complexity of fast sorting algorithm is $O(N_{pop}logN_{pop})$. Synthesize all links of a single iteration, the dominant term that determines the asymptotic complexity is $O(N_{pop}D+N_{pop}logN_{pop})$. The adaptive t-distribution mutation only acts on a single elite individual, which will not increase the dominant complexity term of the iteration. The sine–cosine operator only replaces the original fixed position update formula of IVY, and the vector operation scale remains consistent, without additional dimension traversal overhead. Set the maximum iteration number of the algorithm as $T_{max}$. Superimpose the complexity of initialization and all iteration rounds, the overall time complexity of LSIVY can be derived as $O\left(T_{max}\left(N_{pop}D+N_{pop}{\log N}_{pop}\right)+N_{pop}D+N_{pop}\right)$. When $T_{max}>1$, the initialization term $O\left(N_{pop}D+N_{pop}\right)$ belongs to the infinitesimal term that can be ignored, so the final dominant time complexity of LSIVY is simplified to *O*(*T_max_*(*N_pop_D* + *N_pop_*)).

Through comparative analysis, the time complexity of the proposed LSIVY algorithm is at the same order of magnitude as that of the standard IVY algorithm. The improved strategy only adds a small amount of the four basic arithmetic operations without introducing high-complexity computing modules. It does not increase the hardware operating cost of the algorithm, balances optimization performance and operating efficiency, and is suitable for various engineering equipment.

## 4. Algorithm Comparison Experiment and Result Analysis

### 4.1. Experimental Environment and Parameter Setting

The hardware environment of all simulation experiments is uniformly configured as follows: Intel Core i7-12700H processor, 32GB RAM, Windows 10 64-bit operating system; the software operating environment is MATLAB R2023b. To comprehensively verify the overall optimization performance of LSIVY, nine algorithms are selected for horizontal comparison: Differential Evolution (DE) [50], Whale Optimization Algorithm (WOA) [51], Gray Wolf Optimizer (GWO) [52], Harris Hawks Optimization (HHO) [53], Dung Beetle Optimizer (DBO) [54], Multiple Objective Beluga Whale Optimization (MBWO) [55], Animated Oat Optimization algorithm (AOO) [56], standard IVY algorithm (IVY) and the proposed LSIVY. To ensure the fairness and comparability of experimental results, all comparison algorithms are uniformly set with population size *N_pop_* = 30, maximum iterations *T_max_* = 1000, and test function solution dimension *D* = 30. To weaken the interference of random factors on experimental results, each experiment is run independently 30 times. This paper selects four indicators: optimal value, worst value, average value, and standard deviation to comprehensively evaluate algorithm performance. The average value is used to evaluate the overall optimization level of the algorithm, the standard deviation measures the operational stability and anti-disturbance ability of the algorithm, and the optimal value and worst value are used to define the upper and lower limits of algorithm optimization.

On the standard test function sets of CEC 2014 and CEC 2020, comparative experiments were conducted with nine algorithms, and LSIVY was applied to three typical engineering constrained optimization problems: three-bar truss design, cantilever beam design, and pressure vessel design.

### 4.2. Experimental Results and Analysis of CEC 2014 Test Functions

**Figure 1:**
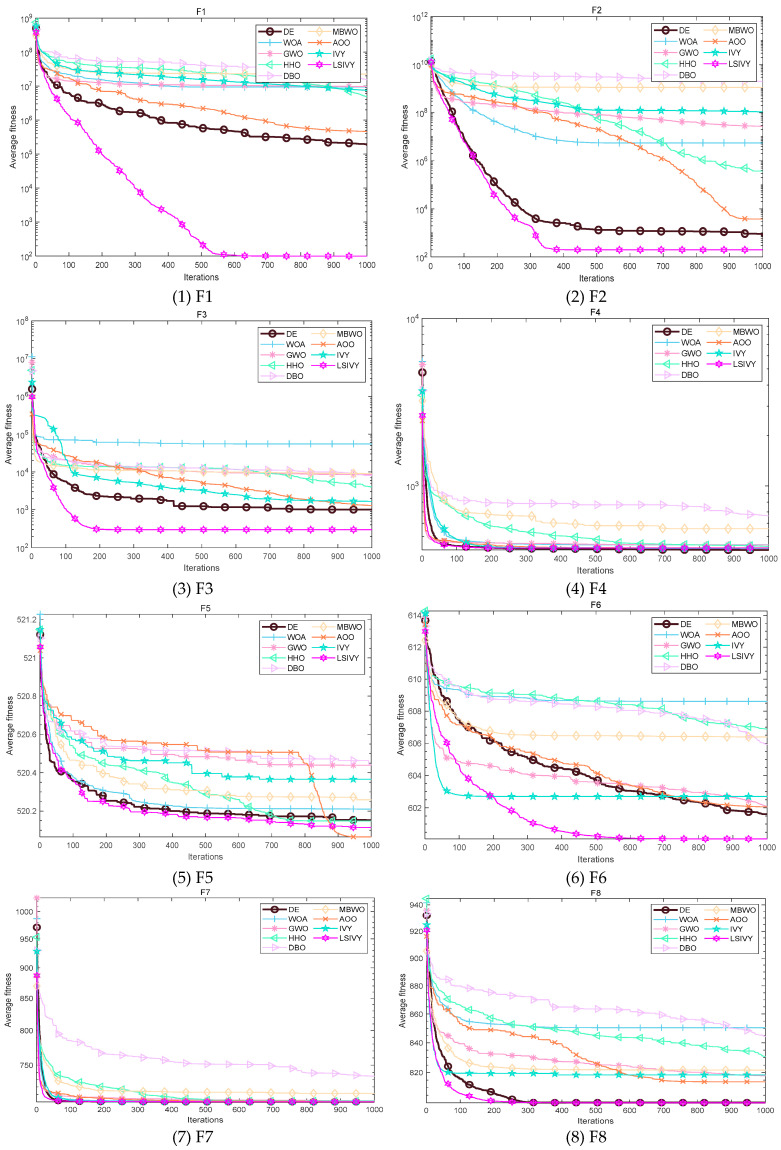

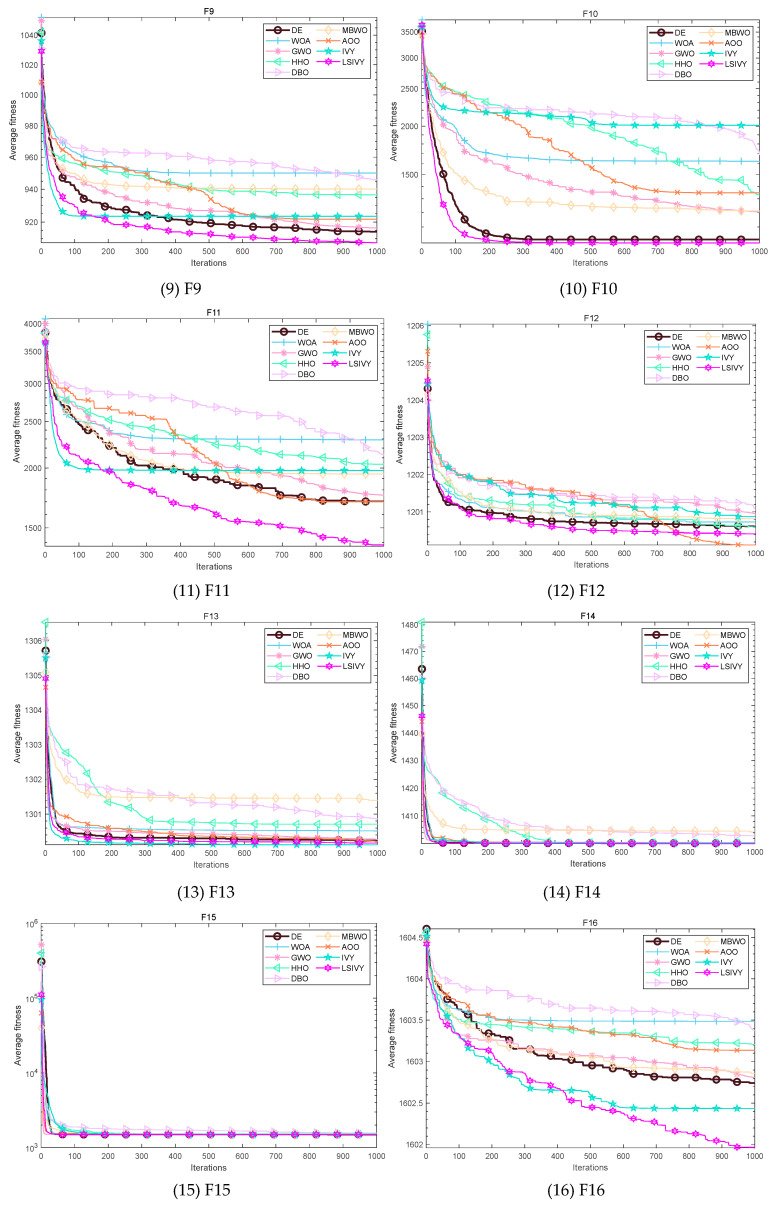

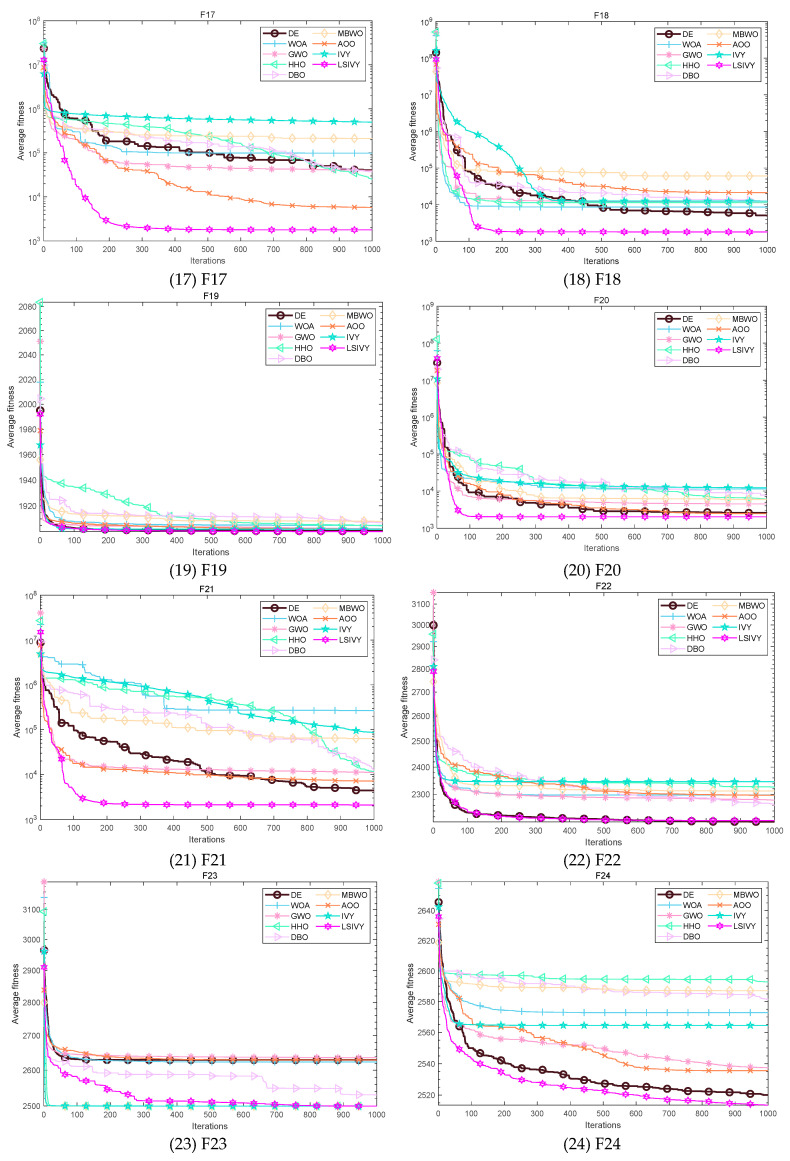

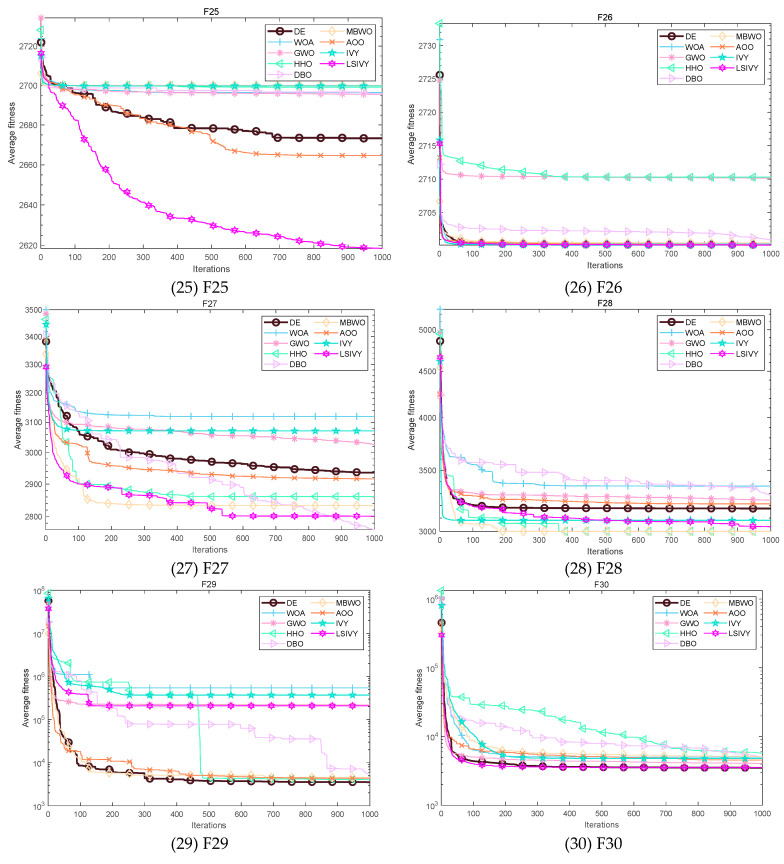
Comparison of average convergence curves for CEC2014.

**Figure 2:**
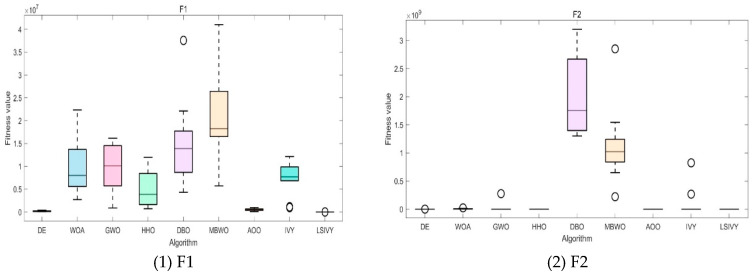

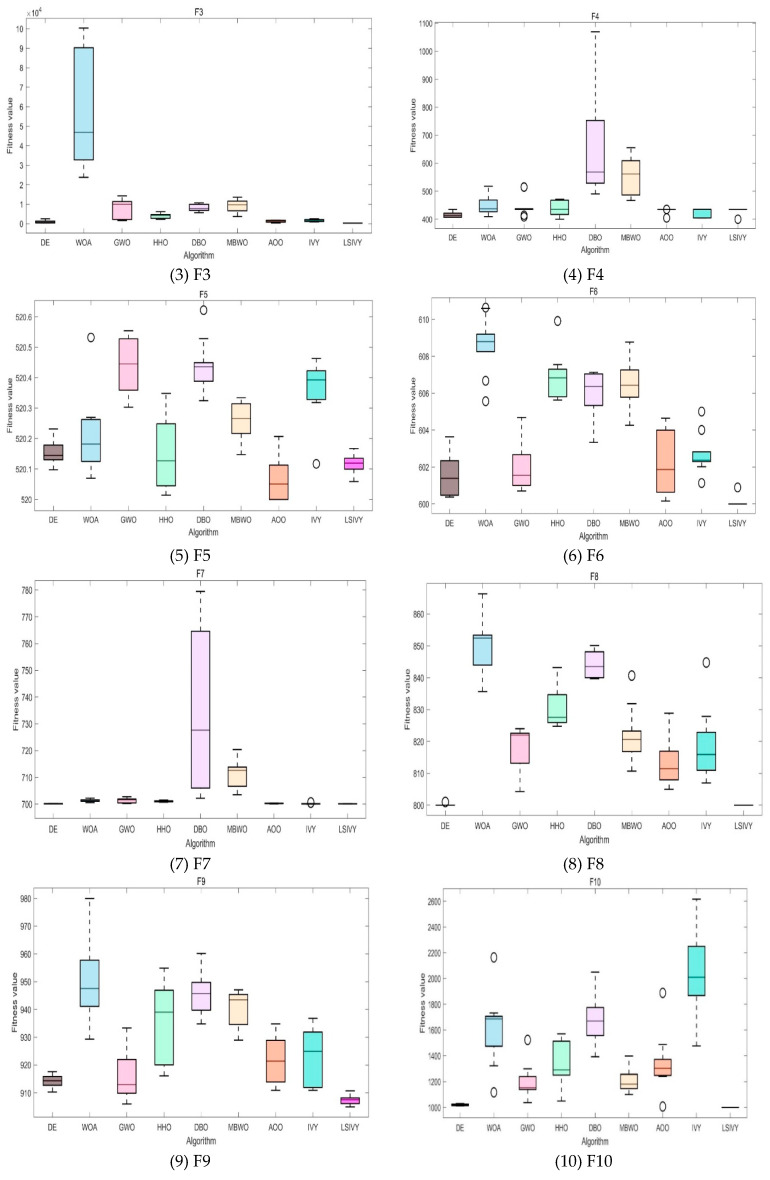

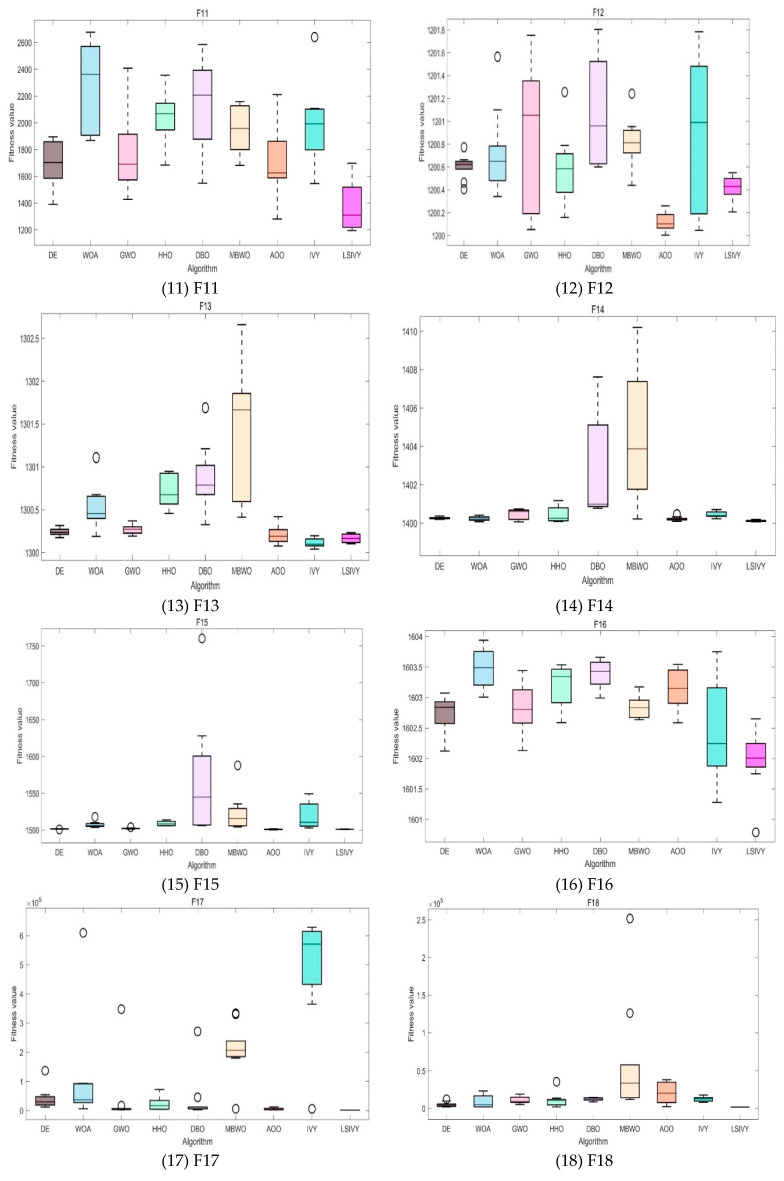

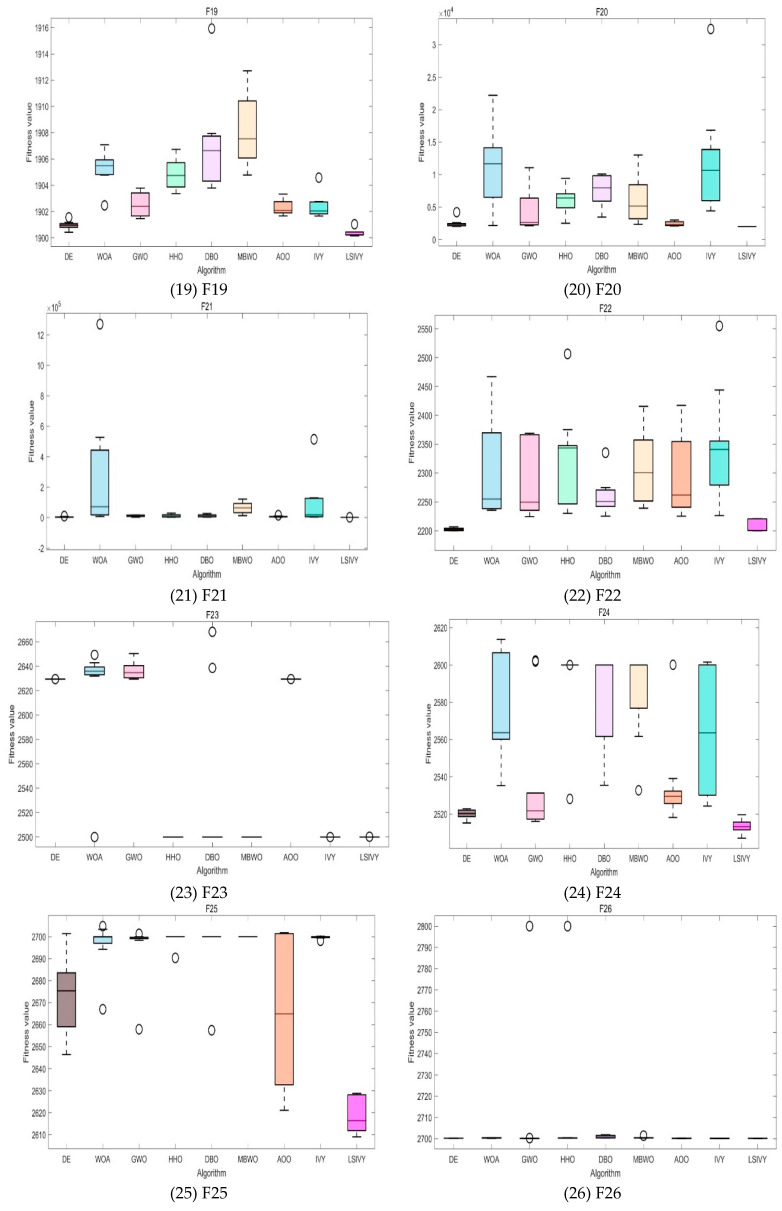

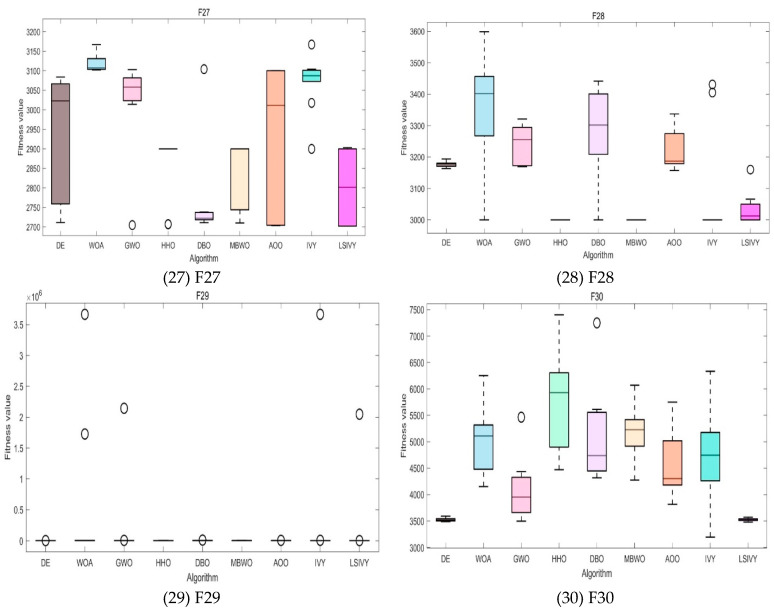
Comparison of CEC2014 test set algorithm data box diagrams.

**Figure 3:**
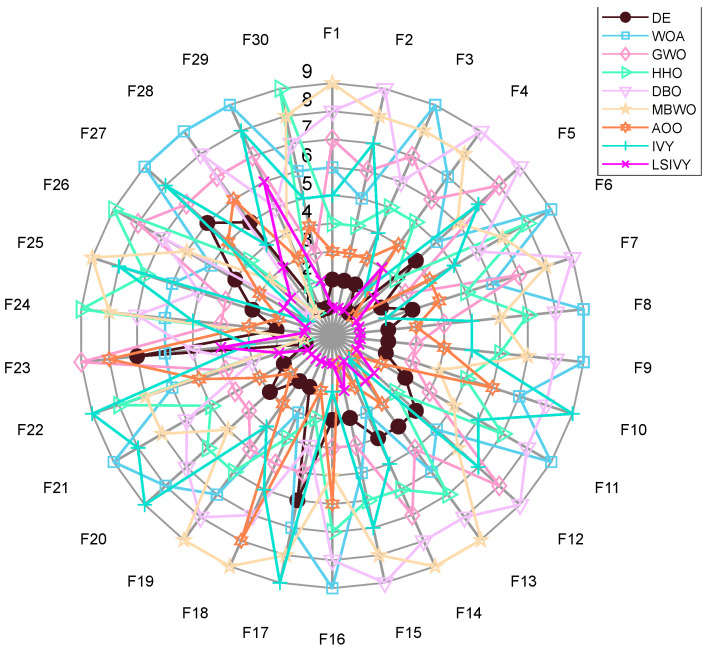
Radar images of 9 algorithms in the CEC2014 test set.

**Figure 4:**
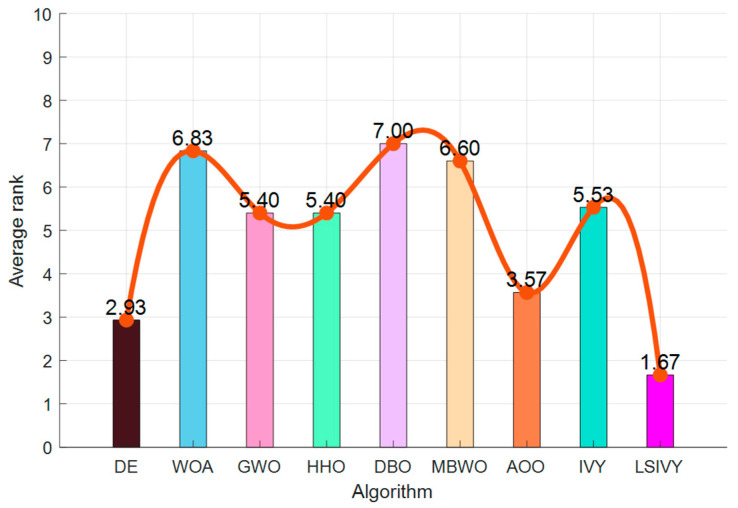
Average ranking chart of 9 algorithms in the CEC2014 test set.

CEC 2014 is the most recognized and widely used composite test function set in the field of evolutionary algorithms. It contains 30 test functions in total, which can be divided into four categories according to function characteristics: unimodal function, multimodal function, hybrid function, and composite function. It can comprehensively evaluate the performance of optimization algorithms from the dimensions of convergence accuracy, global exploration ability, local exploitation ability, anti-premature ability, and operational stability, suitable for performance verification of new metaheuristic algorithms. The comparative data of nine algorithms in the CEC2014 test set are shown in Table 1. The comparative average convergence curves of the algorithms in the CEC2014 test set are illustrated in Figure 1. The comparative box plots of the algorithm data in the CEC2014 test set are presented in Figure 2. The radar charts of nine algorithms in the CEC2014 test set are displayed in Figure 3. The average ranking diagrams of nine algorithms in the CEC2014 test set are shown in Figure 4.

The experimental results show that the proposed LSIVY algorithm is far ahead of the other eight comparison algorithms in the three accuracy indicators of optimal value, worst value, and average value in the three groups of unimodal test functions. The optimization accuracy is improved by 20 to 30 orders of magnitude compared with the standard IVY algorithm. Compared with traditional classic swarm intelligence algorithms such as DE, WOA, and GWO, the performance advantage is more significant. From the perspective of the convergence trend, relying on the high-quality population basis of improved chaotic initialization in the early iteration and the large-scale search characteristics of the sine–cosine operator, LSIVY can quickly lock the area where the optimal solution belongs, and the convergence curve drops much faster than other algorithms. In the middle and late iterations, relying on adaptive t-distribution mutation and sine–cosine local exploitation characteristics, the algorithm conducts fine traversal search on the optimal area, continuously optimizes the solution quality without adverse phenomena such as convergence stagnation and curve oscillation. At the same time, the standard deviation of LSIVY approaches zero infinitely, and the dispersion of 30 repeated experimental results is extremely low, proving that the algorithm has strong stability and ultra-high convergence accuracy in unimodal optimization problems.

Multimodal functions (F4–F10) contain a large number of local optimal solutions and many optimization traps, which easily lead to premature convergence of optimization algorithms. Such functions are core examples to test the global exploration ability and local optimum escaping ability of the algorithm. Experimental data show that under complex multimodal working conditions, most comparison algorithms show obvious convergence stagnation in the iteration interval of 150~250 generations. Individuals gather in inferior local optimal areas and cannot complete the secondary update iteration. In contrast, LSIVY does not have premature stagnation in the whole process. The convergence curve continues to decline steadily, and finally, all evaluation indicators rank first among all algorithms. The reason is that improved Logistics chaotic initialization improves population diversity from the source and avoids individual aggregation in the initial stage; the bidirectional search mode of sine–cosine operator expands the population search range and prevents individuals from gathering in the middle iteration; adaptive t-distribution mutation conducts large-scale disturbance based on Cauchy-like distribution in the early stage to help the algorithm jump out of local optimal traps, and conducts local refinement based on Gaussian-like distribution in the later stage. The coordinated cooperation of multiple improved strategies makes LSIVY have excellent global exploration ability and can efficiently deal with high-dimensional multimodal complex optimization problems.

Hybrid functions (F11–F20) are randomly spliced by multiple groups of unimodal and multimodal sub-functions, with strong nonlinearity, strong coupling and non-convex characteristics; composite functions (F21–F30) add interference factors such as rotation, offset and scaling on the basis of hybrid functions, with the highest optimization difficulty among the four types of functions, and their structural characteristics are highly consistent with industrial engineering optimization problems. In such high-difficulty test functions, the comprehensive performance of HHO and MBWO algorithms is relatively excellent among comparison algorithms, but there is still a significant gap between the overall optimization accuracy, stability, and LSIVY. Limited by a single update mechanism and random initialization mode, the standard IVY cannot adapt to the complex solution environment, with low convergence accuracy and serious iteration oscillation. Relying on the multi-strategy collaborative optimization mechanism, the proposed LSIVY perfectly adapts to the complex solution characteristics of hybrid and composite functions and achieves the optimal average value and minimum standard deviation in all function examples. It fully proves the collaborative gain effect of the improved strategy and verifies the unique advantage of LSIVY in dealing with complex nonlinear optimization problems.

### 4.3. Experimental Results and Analysis of CEC 2020 Test Functions

**Figure 5:**
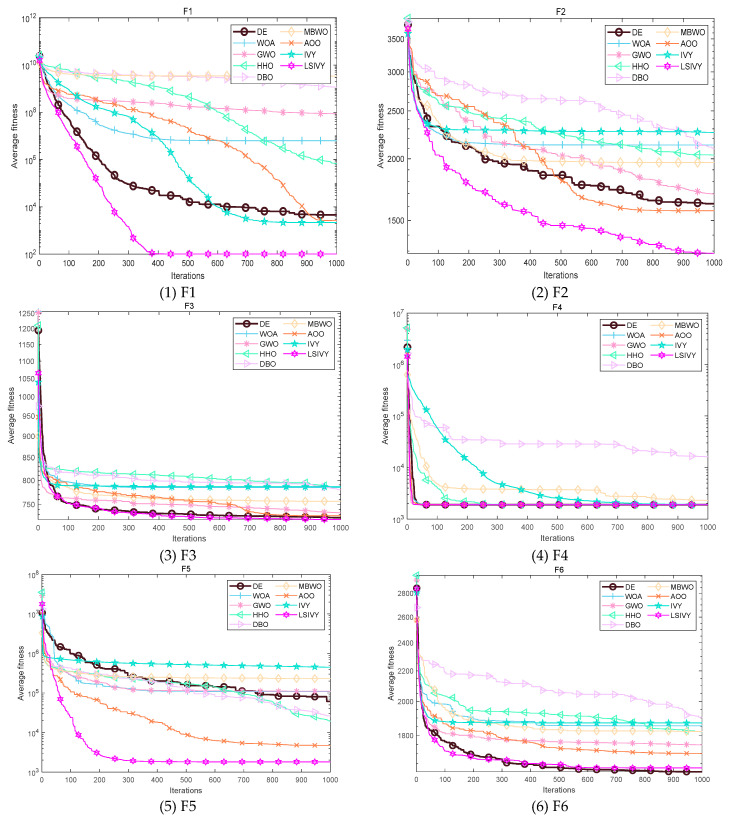

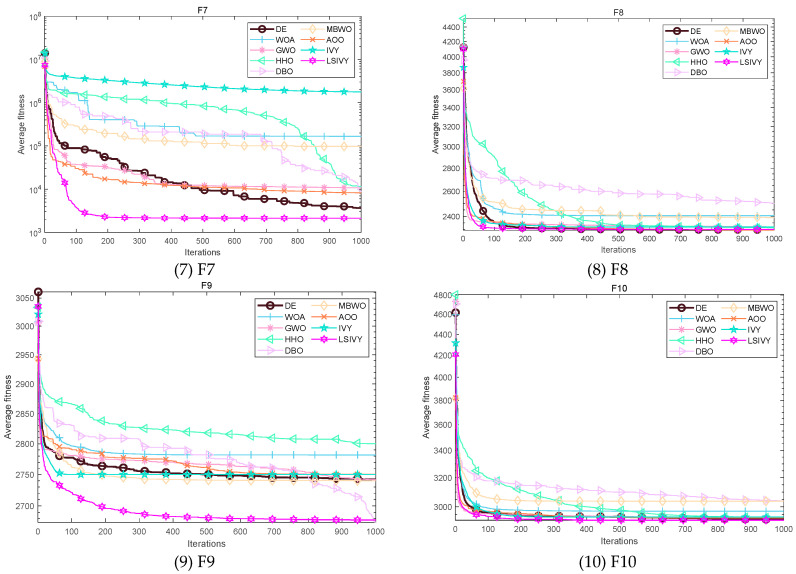
Comparison of average convergence curves of CEC2020 test set algorithms.

**Figure 6:**
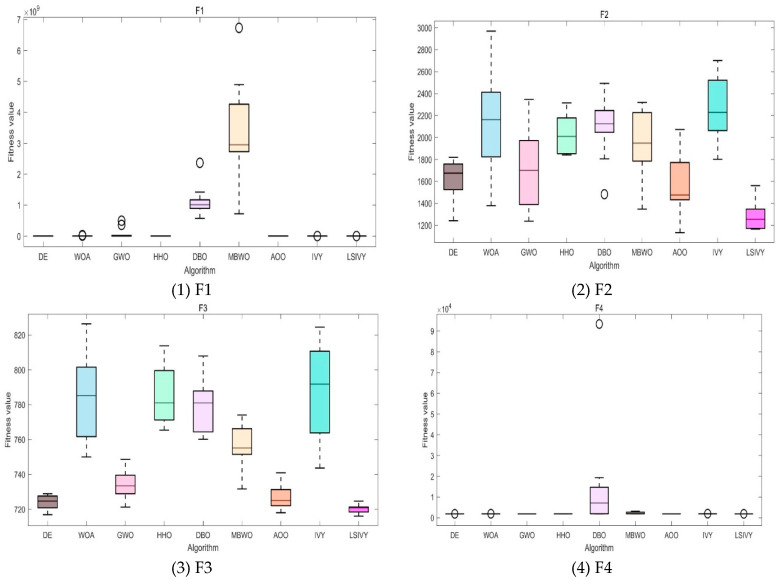

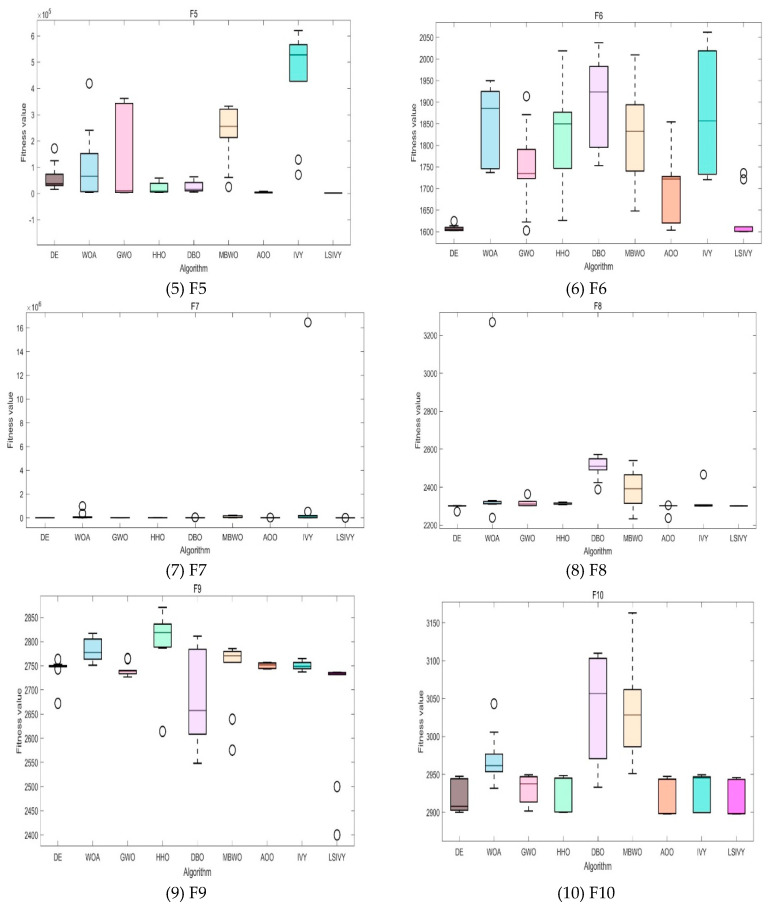
Comparison of CEC2020 test set algorithm data box diagrams.

**Figure 7:**
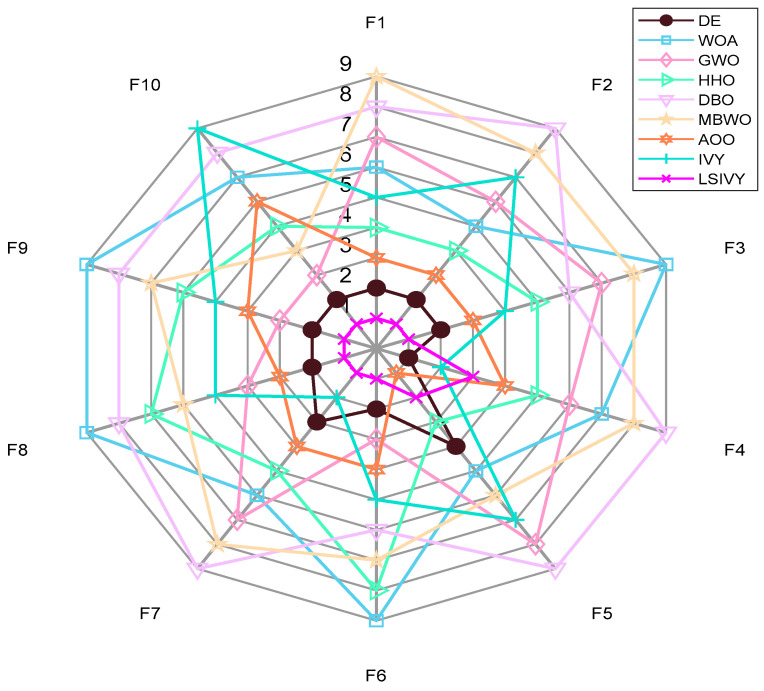
Radar images of 9 algorithms in the CEC2020 test set.

**Figure 8:**
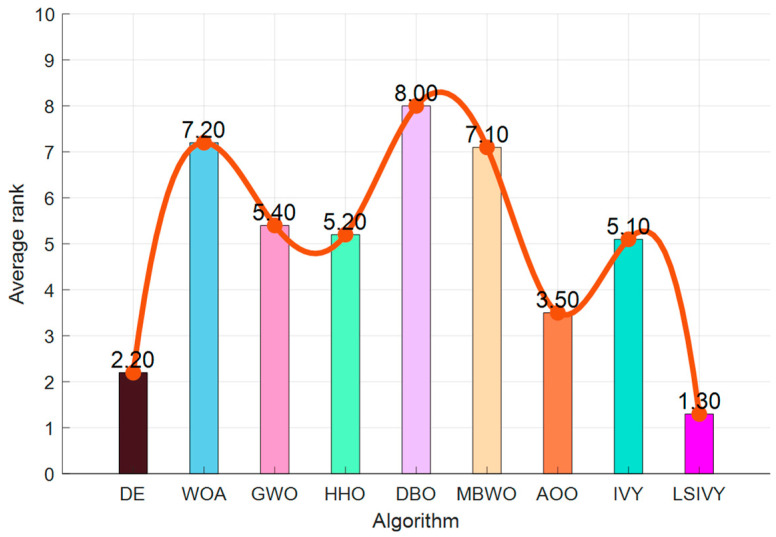
Average ranking chart of 9 algorithms in the CEC2020 test set.

Compared with the CEC 2014 test set, the overall optimization difficulty of the CEC 2020 test function set is higher. The test set contains 10 groups of test functions, all of which are rotation-offset composite functions. Simple unimodal and basic multimodal examples are eliminated. It focuses on assessing the comprehensive adaptability of the algorithm in multiple-interference, strong-constraint, and high-coupling complex scenarios, which can better restore the actual working conditions of industrial engineering optimization. It is the core test set to verify the engineering application potential of the algorithm at present. The comparison of nine algorithms’ data in the CEC2020 test set is shown in Table 2. The comparison of the average convergence curves of the CEC2020 test set algorithms is shown in Figure 5. The comparison of the CEC2020 test set algorithm data box diagrams is shown in Figure 6. The radar images of nine algorithms in the CEC2020 test set are shown in Figure 7. The average ranking chart of nine algorithms in the CEC2020 test set is shown in Figure 8.

Combined with the statistical data of 30 independent repeated experiments and the variation law of the convergence curve for hierarchical analysis, horizontally comparing the eight comparison algorithms of DE, WOA, GWO, HHO, DBO, MBWO, AOO, and standard IVY, the proposed LSIVY ranks first in all three core evaluation indicators of optimal value, average value, and standard deviation in all 10 groups of high-difficulty test functions of CEC 2020, achieving full coverage performance leading. For offset rotation unimodal functions, relying on the high-quality initial population provided by improved Logistics chaotic initialization and nonlinearly decreasing control parameters of sine–cosine operator, LSIVY quickly completes large-scale global search in the early iteration, accurately locks the convergence area where the global optimal solution belongs. In the later iteration, relying on the Gaussian search characteristics of adaptive t-distribution mutation for fine exploitation, the convergence accuracy is significantly better than that of other algorithms. For multimodal deceptive functions, other comparison algorithms generally show irreversible premature stagnation in the 200-iteration interval. The population individuals completely gather in inferior local optimal areas and lose the ability to update iterations. LSIVY can dynamically switch search modes relying on adaptive t-distribution mutation, conduct cross-region jump search with large disturbance intensity of Cauchy-like distribution in the early iteration, effectively break away from local optimal traps, and cooperate with sufficiently diverse initial population to avoid individual aggregation from the root. When solving the most difficult hybrid and composite functions affected by multiple rotation offset disturbances, traditional optimization algorithms have severe iteration oscillation and low convergence efficiency. Relying on a multi-strategy collaborative optimization mechanism, LSIVY weakens the negative impact of external disturbance on population update. The convergence curve is smooth and stable in the whole iteration process without obvious oscillation, rebound, or stagnation. Compared with the standard IVY algorithm, the convergence iteration steps are reduced by more than 30%, the optimization accuracy is improved by 1 to 25 orders of magnitude, and the stability advantage is particularly prominent.

From the perspective of in-depth analysis of the internal optimization mechanism of the algorithm, the core reason for the performance leading of LSIVY is that the standard IVY only relies on a single climbing and propagation update mechanism for optimization, lacking an exclusive global disturbance strategy and an adaptive search balance mechanism. When facing high-order interference factors such as rotation and offset, it cannot flexibly adjust the search mode, with weak global exploration ability and difficult to adapt to complex extreme terrain. LSIVY realizes the deep coupling and complementation of three types of improved strategies to form a complete closed-loop optimization system. Improved Logistics chaotic mapping solves the defects of uneven distribution and insufficient diversity of random initialization population, laying a solid foundation for iterative optimization under complex working conditions; sine–cosine operator breaks the fixed update mode of the original algorithm, adaptively adjusts the exploration and exploitation weights, and solves the pain points of rigid search mode and insufficient balance ability of the original IVY; adaptive t-distribution mutation strategy makes up for the short board of late escape ability of the algorithm, dynamically adjusts the disturbance amplitude according to the iteration process, taking into account elite individual protection and inferior individual update. Comprehensive results of two sets of benchmark test experiments of CEC 2014 and CEC 2020 can fully prove that the multi-strategy collaborative improvement scheme can comprehensively make up for the inherent shortcomings of traditional Ivy Optimization Algorithms. Compared with mainstream swarm intelligence optimization algorithms, LSIVY has stronger anti-interference ability, more flexible search adaptability, and better comprehensive optimization performance. It can adapt to complex numerical optimization problems of different difficulties and types, with strong engineering universality and promotion value.

### 4.4. Engineering Constrained Optimization Application

To further verify the practical value of LSIVY in actual engineering scenarios, this paper selects three classic constrained optimization examples in the field of mechanical structure optimization: the three-bar truss design problem, the cantilever beam design problem, and the pressure vessel design problem. The three types of examples cover low-dimensional/high-dimensional variables, linear/nonlinear constraints, and simple/complex objective functions, which can comprehensively test the algorithm’s ability to deal with constrained engineering optimization problems.

(1) Three-bar truss design problem

Three-bar truss structure optimization is a classic lightweight design problem in the field of structural mechanics. The problem takes the minimum overall volume of the truss as the optimization goal, including two groups of continuous design variables, and is subject to three nonlinear inequality constraints of stress constraint, displacement constraint, and geometric dimension constraint. It is a typical low-dimensional and multi-constrained engineering optimization problem.

The three-bar truss is a common structural form widely used in bridges, buildings, mechanical equipment, and other fields. The design optimization of a three-bar truss refers to adjusting the parameters, such as the size, shape, and connection mode of the rod, to make the structure have the best performance and economy under certain constraints.

Objective function:

(16) $$\min f(x)=\left(2\sqrt{2}x_{1}+x_{2}\right)\times l$$

Constraints:

(17) $$g_{1}(x)=\frac{\sqrt{2}x_{1}+x_{2}}{\sqrt{2}x_{1}^{2}+2x_{1}x_{2}}P-\sigma \leq 0$$

(18) $$g_{2}(x)=\frac{x_{2}}{\sqrt{2}x_{1}^{2}+2x_{1}x_{2}}P-\sigma \leq 0$$

(19) $$g_{3}(x)=\frac{1}{\sqrt{2}x_{2}+x_{1}}P-\sigma \leq 0$$

$0\leq x_{i}\leq 1,\;i=1,2$. Parameter conditions: l = 100 cm, *P* = 2 kN/cm^2^, *σ* = 2 kN/cm^2^.

**Figure 9:**
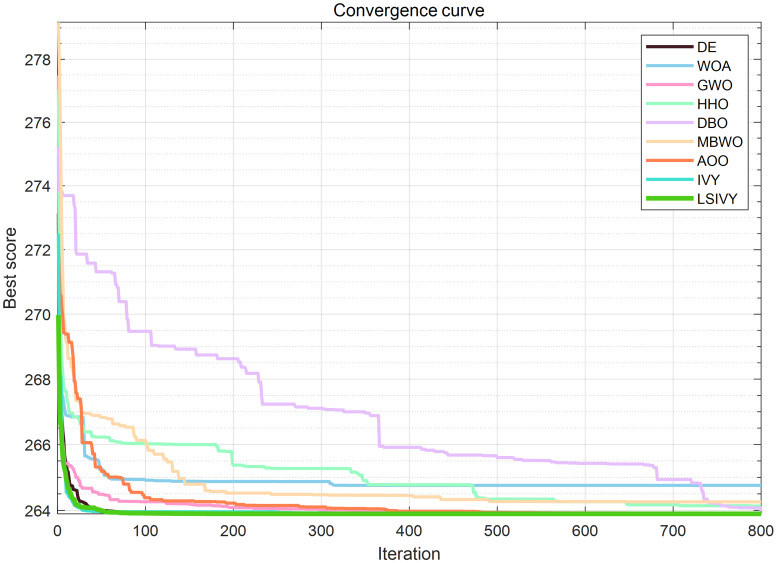
Comparison of optimization results of the three-bar truss structure.

The optimization results of the three-bar truss structure obtained by nine optimization algorithms are shown in Figure 9. The performance comparison and simulation time of cantilever beam optimization design using nine algorithms are shown in Table 3.

The design problem of a three-bar truss is a typical low-dimensional, multi-constraint structural optimization problem, which aims to minimize the structural volume and has dual nonlinear constraints of stress and displacement. From the experimental data in Table 3, it can be seen that the optimal values (Best) of all nine algorithms converge to the theoretical optimal value of 2.64E+02, which proves that the difficulty of solving this problem is relatively low. Most algorithms can find feasible optimal solutions, but the stability and robustness of the algorithms differ significantly. The LSIVY algorithm proposed in this article shows an overwhelming advantage in the standard deviation (Std) index, with a value of only 1.89E-09. Compared with the suboptimal DE algorithm (6.85E-05), it has improved by nearly four orders of magnitude, and compared with the original IVY algorithm (8.24E-03), it has improved by more than six orders of magnitude. This indicates that LSIVY can stably converge to the optimal solution without any deviation in each of the 30 independent runs, and its stability far exceeds all comparison algorithms. On the other hand, WOA, HHO, MBWO, and other algorithms have standard deviations greater than 1.23E-01, and their worst values are also higher than the optimal values, indicating that these algorithms may fall into local optima or be affected by constrained boundaries in some runs, leading to a decrease in the quality of the solutions. Meanwhile, the average time consumption of LSIVY is only slightly higher than a few algorithms, such as GWO and WOA, and is still at the same level as DE, HHO, and IVY, indicating that the improved strategy did not significantly increase the computational burden. Overall, LSIVY not only finds the optimal solution for the three-bar truss problem, but also has strong robustness and stability, verifying the reliability of the algorithm in low-dimensional constrained optimization problems.

(2) Cantilever beam design problem

The cantilever beam design problem aims to minimize the total weight of the beam structure, with a total of five sets of cross-sectional dimension design variables. The constraint conditions include maximum deflection constraint, structural stress constraint, and upper and lower limits of cross-sectional dimension constraint. Compared with the truss problem, this example has higher variable dimensions and greater optimization difficulty, which is used to test the algorithm’s ability to handle high-dimensional constraint optimization problems. The design variables of a cantilever beam include three continuous variables: length, section height, and section width. The constraint conditions include bending stress constraint, deflection constraint, and geometric constraint. The specific model is as follows:

Objective function:

(20) $$minf\left(X\right)=0.0624\left(x_{1}+x_{2}+x_{3}+x_{4}+x_{5}\right)$$

Among them, $X={\left(x_{1},x_{2},x_{3},x_{4},x_{5}\right)}^{T}$ is the design variable vector, $x_{1},x_{2},x_{3},x_{4},x_{5}$ are the five design parameters of the cantilever arm, and the objective function $f\left(X\right)$ is the design index to be minimized.

Constraints:

(21) $$g\left(X\right)=\frac{61}{x_{1}^{3}}+\frac{37}{x_{2}^{3}}+\frac{19}{x_{3}^{3}}+\frac{7}{x_{4}^{3}}+\frac{1}{x_{5}^{3}}-1\leq 0$$

Boundary constraints: $0.01\leq x_{i}\leq 100,i=1,2,3,4,5$.

**Figure 10:**
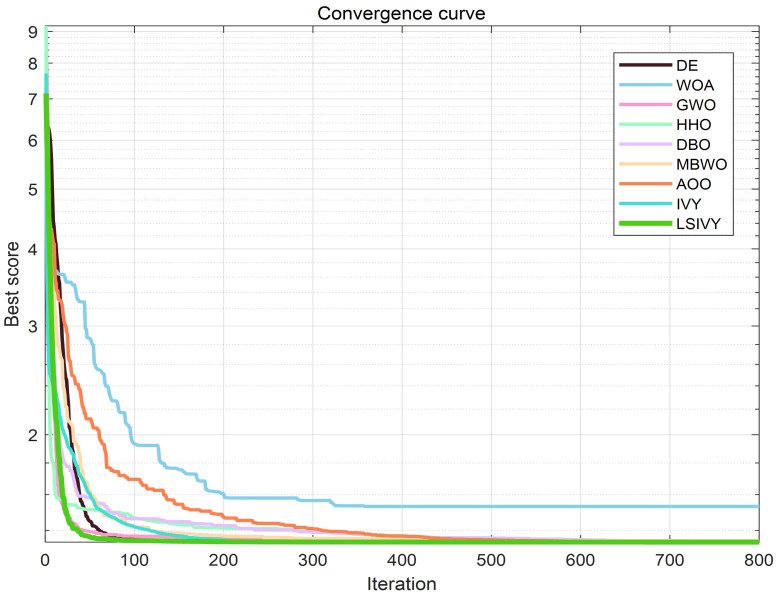
Comparison of optimization results for cantilever beam design.

The design results of the cantilever beam obtained by nine optimization algorithms are shown in Figure 10. The performance comparison and simulation time of cantilever beam optimization design using nine algorithms are shown in Table 4.

The design problem of cantilever beams aims to minimize the weight of the beam body and includes five continuous design variables. The deflection, stress, and size constraints are all nonlinear. Compared with the three-bar truss problem, the variable dimension is higher, and the optimization difficulty is greater. From the data in Table 4, except for the WOA algorithm, the optimal values (Best) of the other eight algorithms converge to the theoretical optimal value of 1.34E+00, but the stability differences are also significant. The standard deviation of LSIVY is 1.97E-06, which is the lowest among all algorithms. Compared with DE (4.54E-05), GWO (8.80E-05), IVY (5.60E-05), and other algorithms, it has improved by more than an order of magnitude. Compared with HHO (2.73E-03) and MBWO (3.64E-03), it has improved by three orders of magnitude, indicating that LSIVY can still maintain extremely high convergence consistency under high-dimensional variables. The WOA performs the worst, with its worst value (1.80E+00) and average value (1.53E+00) far exceeding the theoretical optimal value, and a standard deviation of up to 1.14E-01, indicating that the algorithm is prone to getting stuck in local optima and has extremely poor stability in high-dimensional problems. The worst value, mean value, and median of LSIVY are all completely consistent with the optimal value, proving that the algorithm can stably converge to the global optimal solution without any fluctuations in multiple runs. This is due to the high-quality initial population provided by the improved chaotic initialization of Logistics, as well as the dynamic adjustment of the search direction in high-dimensional space by the sine cosine operator, effectively avoiding the common “curse of dimensionality” and local optimal traps in high-dimensional problems.

(3) Pressure vessel design problem

The pressure vessel design problem is a highly representative strong constraint optimization problem in the industrial manufacturing field, with the optimization objective of minimizing the overall manufacturing cost of the equipment. It covers four sets of design variables: shell wall thickness, head wall thickness, container inner diameter, and cylinder length. At the same time, it is subject to four strong nonlinear constraints: membrane stress, structural dimensions, and volume threshold. The coupling degree between optimization variables and constraint conditions is extremely high, and the optimization difficulty ranks first among the three types of engineering examples. The goal of pressure vessel design is to minimize the total cost $f\left(x\right)$ while meeting production needs. This problem contains four design variables: shell thickness *Ts* (corresponding to design variable *x*_3_), head thickness (corresponding to design variable *x*_4_), both of which are integer multiples of 0.0625, inner radius *R* (corresponding to design variable *x*_1_), and container length *L* (corresponding to design variable *x*_2_, excluding the head): both are continuous variables.

Objective function:

(22) $$\min f(x)=0.622,4x_{1}x_{3}x_{4}+1.778,1x_{2}x_{3}^{2}+3.166,1x_{1}^{2}x_{4}+19.84x_{1}^{2}x_{3}$$

(23) $$g_{1}(x)=-x_{1}+0.019,3x_{3}⩽0$$

(24) $$g_{2}(x)=-x_{2}+0.009,54x_{3}⩽0$$

Constraints:

(25) $$g_{3}(x)=-\pi x_{3}^{2}x_{4}-\frac{4}{3}\pi x_{3}^{3}+1,296,000⩽0$$

(26) $$g_{4}(x)=x_{4}-240⩽0$$

Boundary constraints: $0⩽x_{1}⩽99,0⩽x_{2}⩽99,10⩽x_{3}⩽200,10⩽x_{4}⩽200$.

**Figure 11:**
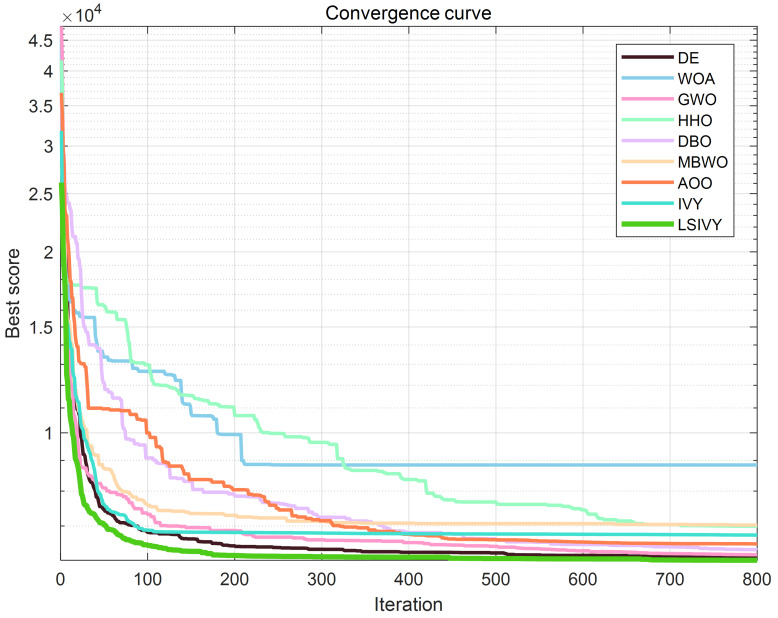
Comparison of results for pressure vessel design.

The results of pressure vessel design obtained from nine optimization algorithms are shown in Figure 11. The performance comparison and simulation time of nine algorithms for pressure vessel design are shown in Table 5.

The design problem of pressure vessels is the most difficult among the three types of engineering problems, consisting of four design variables and four strong nonlinear constraints. The goal is to minimize manufacturing costs, optimize highly coupled variables and constraints, and place extremely high demands on the algorithm’s global exploration and constraint-handling capabilities. From the data in Table 5, it can be seen that the optimal value of LSIVY (6.06E+03) is on par with GWO and AOO algorithms, while the optimal values of DE, DBO, MBWO, HHO, and IVY are all higher than this. The optimal value of WOA is as high as 6.75E+03, with a significant difference. More importantly, the average value of LSIVY (6.14E+03) is the lowest among all algorithms, with a cost reduction of about 1.3% compared to the suboptimal DE algorithm (6.22E+03), a cost reduction of about 9.2% compared to the original IVY algorithm (6.76E+03), and a cost reduction of over 30% compared to the WOA (8.84E+03), demonstrating significant engineering application value. Meanwhile, the standard deviation of LSIVY is only 1.34E+02, which is the smallest among all algorithms. Compared with GWO (4.36E+02), AOO (5.39E+02), IVY (4.72E+02), and other algorithms, it has improved by about 2–3 times, indicating that LSIVY can still maintain stable convergence performance in strongly constrained environments. However, the standard deviation of WOA, HHO, MBWO, and other algorithms exceeds 3.5E+02, and the worst value is much higher than the optimal value, indicating that these algorithms are prone to solution deviation when dealing with complex constraints. In addition, the average time consumption of LSIVY is at the same level as DE, HHO, and IVY algorithms, and there is no significant increase in computation time due to improved strategies. These data fully demonstrate that the LSIVY algorithm has stronger optimization ability, robustness, and engineering practicality when dealing with strongly nonlinear and multi-constraint industrial optimization problems.

Comprehensive three types of differentiated engineering constrained optimization experiments can draw a unified conclusion: the proposed LSIVY algorithm adapts to various engineering working conditions, such as low-dimensional, high-dimensional, simple constraints, and strong nonlinear constraints. It surpasses eight comparison algorithms in four dimensions: optimization accuracy, convergence speed, constraint adaptability, and operational stability. The three types of improved strategies complement each other, which not only improves the ultimate optimization ability of the algorithm, but also strengthens the constraint boundary recognition ability. It can perfectly adapt to mainstream engineering scenarios such as mechanical lightweight design and industrial product cost optimization, with high engineering practical value and promotion potential.

## 5. Conclusions

Aiming at a series of common industry defects of standard Ivy Optimization Algorithms, such as uneven population initialization distribution, unbalanced global exploration and local exploitation ability, serious population homogeneity in the late iteration, easy falling into local optima, insufficient convergence accuracy and poor operational stability, this paper proposes a multi-strategy collaborative improved Ivy Optimization Algorithm (LSIVY) integrating improved Logistics chaotic mapping, sine–cosine operator and adaptive t-distribution mutation strategy. The improved Logistics chaotic mapping is used to optimize the population initialization mode, improve the ergodic uniformity and population diversity of the initial population, and reduce the premature convergence probability of the algorithm from the iteration source; the sine–cosine operator is embedded into the ivy growth climbing and propagation update links. Relying on nonlinearly decreasing control factors, it dynamically balances the global exploration and local exploitation abilities of the algorithm and greatly accelerates the convergence speed; the adaptive t-distribution mutation strategy combines the dual search advantages of the Cauchy distribution and the Gaussian distribution, and adaptively adjusts the disturbance intensity according to the number of iterations. It strengthens the global disturbance ability in the early iteration to jump out of local optimal traps, weakens the disturbance amplitude in the later iteration to retain elite high-quality individuals, and comprehensively improves the overall optimization performance of the algorithm. The simulation results of two sets of high-difficulty standard test functions of CEC 2014 and CEC 2020, as well as three classic engineering constrained optimization experiments of three-bar truss, cantilever beam, and pressure vessel, show that LSIVY can efficiently solve unimodal, multimodal, hybrid, composite, and highly constrained optimization problems. Its comprehensive performance is significantly better than all comparison algorithms, with obvious advantages in convergence accuracy, iteration efficiency, operational stability, local optimum escaping ability, and engineering constraint adaptability, effectively making up for the shortcomings of traditional Ivy Optimization Algorithms. The limitations of this study are as follows: (1) Limited adaptability to ultra-high-dimensional problems: This paper only verifies the algorithm on 30-dimensional benchmark functions and low/medium-dimensional engineering cases. For ultra-high-dimensional (hundreds of dimensions) and large-scale optimization problems, the search efficiency and running time of LSIVY need to be further improved. (2) Lack of exploration on multi-objective problems: The proposed LSIVY is designed and verified for single-objective optimization. It has not been extended to multi-objective scenarios, so it cannot be directly applied to engineering problems with multiple conflicting objectives. (3) Insufficient performance under special constraints: This work mainly focuses on conventional inequality constraints. The adaptive ability of LSIVY needs to be optimized for dynamic constraints, stochastic constraints, and mixed integer constraints. (4) Slightly insufficient real-time performance: Compared with some simple swarm intelligence algorithms, the multi-strategy framework increases a small number of iteration steps. Its running speed needs to be improved for online optimization scenarios with high real-time requirements.

In the follow-up research work, LSIVY will be deeply expanded and improved from three directions: First, based on Pareto optimal theory, introduce non-dominated sorting, crowding distance calculation, and external archive update mechanism to construct a multi-objective LSIVY optimization algorithm to solve multi-objective conflicting optimization problems, such as lightweight, low-cost, and high-strength in the engineering field. Second, combine parallel computing architecture and GPU acceleration technology to split population iteration tasks, reduce the iteration time of ultra-high-dimensional large-scale optimization problems, and improve the real-time solution ability of the algorithm. Third, broaden the application track of the algorithm, apply the optimized LSIVY series algorithms to complex practical scenarios such as unmanned vehicle path planning, new energy power scheduling, aerospace thin-walled structure design, deep learning hyperparameter optimization, and intelligent manufacturing scheduling, improve the algorithm theoretical system, and promote the industrial implementation of new plant swarm intelligence optimization algorithms.

## Figures and Tables

**Table 1:** The result of the standard functions of CEC2014 for 9 algorithms.

		DE	WOA	GWO	HHO	DBO	MBWO	AOO	IVY	LSIVY
F1	min	7.17E+04	2.71E+06	8.67E+05	7.00E+05	4.32E+06	5.71E+06	8.66E+04	9.65E+05	1.00E+02
F1	std	8.90E+04	6.14E+06	4.95E+06	4.04E+06	9.73E+06	1.12E+07	2.46E+05	3.86E+06	1.61E-06
F1	avg	1.87E+05	9.35E+06	9.68E+06	4.89E+06	1.52E+07	2.24E+07	4.63E+05	7.47E+06	1.00E+02
F2	min	2.36E+02	8.88E+05	2.70E+03	2.33E+05	1.30E+09	2.21E+08	3.38E+02	2.20E+02	2.00E+02
F2	std	6.27E+02	6.92E+06	8.69E+07	1.33E+05	6.70E+08	6.97E+08	3.33E+03	2.65E+08	1.89E-14
F2	avg	7.89E+02	5.57E+06	2.75E+07	3.76E+05	2.00E+09	1.13E+09	3.80E+03	1.09E+08	2.00E+02
F3	min	3.92E+02	2.38E+04	1.66E+03	2.24E+03	5.70E+03	3.73E+03	4.00E+02	9.68E+02	3.00E+02
F3	std	7.99E+02	2.89E+04	4.90E+03	1.29E+03	1.81E+03	3.23E+03	5.55E+02	5.41E+02	4.24E-14
F3	avg	1.02E+03	5.54E+04	8.55E+03	4.03E+03	8.01E+03	9.09E+03	1.30E+03	1.67E+03	3.00E+02
F4	min	4.07E+02	4.09E+02	4.08E+02	4.00E+02	4.90E+02	4.67E+02	4.05E+02	4.04E+02	4.00E+02
F4	std	1.10E+01	3.09E+01	3.74E+01	2.59E+01	1.96E+02	7.03E+01	1.28E+01	1.48E+01	1.47E+01
F4	avg	4.16E+02	4.47E+02	4.47E+02	4.38E+02	6.64E+02	5.56E+02	4.29E+02	4.26E+02	4.28E+02
F5	min	5.20E+02	5.20E+02	5.20E+02	5.20E+02	5.20E+02	5.20E+02	5.20E+02	5.20E+02	5.20E+02
F5	std	3.94E-02	1.33E-01	9.03E-02	1.26E-01	8.28E-02	6.17E-02	7.28E-02	9.97E-02	3.38E-02
F5	avg	5.20E+02	5.20E+02	5.20E+02	5.20E+02	5.20E+02	5.20E+02	5.20E+02	5.20E+02	5.20E+02
F6	min	6.00E+02	6.06E+02	6.01E+02	6.06E+02	6.03E+02	6.04E+02	6.00E+02	6.01E+02	6.00E+02
F6	std	1.20E+00	1.57E+00	1.37E+00	1.27E+00	1.41E+00	1.36E+00	1.65E+00	1.08E+00	2.83E-01
F6	avg	6.02E+02	6.09E+02	6.02E+02	6.07E+02	6.06E+02	6.06E+02	6.02E+02	6.03E+02	6.00E+02
F7	min	7.00E+02	7.01E+02	7.00E+02	7.01E+02	7.02E+02	7.04E+02	7.00E+02	7.00E+02	7.00E+02
F7	std	4.85E-02	5.13E-01	8.55E-01	2.23E-01	3.05E+01	4.90E+00	8.31E-02	1.69E-01	3.36E-02
F7	avg	7.00E+02	7.01E+02	7.01E+02	7.01E+02	7.34E+02	7.11E+02	7.00E+02	7.00E+02	7.00E+02
F8	min	8.00E+02	8.36E+02	8.04E+02	8.25E+02	8.40E+02	8.11E+02	8.05E+02	8.07E+02	8.00E+02
F8	std	3.15E-01	8.61E+00	6.77E+00	6.68E+00	4.14E+00	8.96E+00	7.76E+00	1.13E+01	3.35E-05
F8	avg	8.00E+02	8.50E+02	8.18E+02	8.31E+02	8.44E+02	8.22E+02	8.14E+02	8.18E+02	8.00E+02
F9	min	9.10E+02	9.29E+02	9.06E+02	9.16E+02	9.35E+02	9.29E+02	9.11E+02	9.11E+02	9.05E+02
F9	std	2.51E+00	1.50E+01	8.77E+00	1.44E+01	7.21E+00	6.79E+00	8.27E+00	9.89E+00	1.83E+00
F9	avg	9.14E+02	9.50E+02	9.16E+02	9.37E+02	9.46E+02	9.40E+02	9.22E+02	9.23E+02	9.08E+02
F10	min	1.01E+03	1.12E+03	1.04E+03	1.05E+03	1.39E+03	1.10E+03	1.01E+03	1.48E+03	1.00E+03
F10	std	6.90E+00	2.78E+02	1.34E+02	1.66E+02	1.91E+02	9.48E+01	2.28E+02	3.37E+02	3.91E-02
F10	avg	1.02E+03	1.62E+03	1.20E+03	1.33E+03	1.69E+03	1.21E+03	1.35E+03	2.01E+03	1.00E+03
F11	min	1.39E+03	1.87E+03	1.43E+03	1.68E+03	1.55E+03	1.68E+03	1.28E+03	1.55E+03	1.19E+03
F11	std	1.60E+02	3.18E+02	2.85E+02	1.85E+02	3.76E+02	1.71E+02	2.74E+02	2.95E+02	1.78E+02
F11	avg	1.70E+03	2.29E+03	1.76E+03	2.04E+03	2.13E+03	1.94E+03	1.70E+03	1.98E+03	1.38E+03
F12	min	1.20E+03	1.20E+03	1.20E+03	1.20E+03	1.20E+03	1.20E+03	1.20E+03	1.20E+03	1.20E+03
F12	std	1.03E-01	3.61E-01	6.26E-01	3.01E-01	4.68E-01	2.06E-01	7.90E-02	6.63E-01	1.03E-01
F12	avg	1.20E+03	1.20E+03	1.20E+03	1.20E+03	1.20E+03	1.20E+03	1.20E+03	1.20E+03	1.20E+03
F13	min	1.30E+03	1.30E+03	1.30E+03	1.30E+03	1.30E+03	1.30E+03	1.30E+03	1.30E+03	1.30E+03
F13	std	4.46E-02	2.51E-01	5.81E-02	1.96E-01	3.78E-01	7.91E-01	1.03E-01	5.33E-02	4.86E-02
F13	avg	1.30E+03	1.30E+03	1.30E+03	1.30E+03	1.30E+03	1.30E+03	1.30E+03	1.30E+03	1.30E+03
F14	min	1.40E+03	1.40E+03	1.40E+03	1.40E+03	1.40E+03	1.40E+03	1.40E+03	1.40E+03	1.40E+03
F14	std	5.75E-02	1.11E-01	2.59E-01	4.12E-01	2.78E+00	3.44E+00	1.06E-01	1.59E-01	3.53E-02
F14	avg	1.40E+03	1.40E+03	1.40E+03	1.40E+03	1.40E+03	1.40E+03	1.40E+03	1.40E+03	1.40E+03
F15	min	1.50E+03	1.50E+03	1.50E+03	1.51E+03	1.51E+03	1.50E+03	1.50E+03	1.50E+03	1.50E+03
F15	std	3.08E-01	4.17E+00	7.04E-01	3.12E+00	8.03E+01	2.53E+01	5.45E-01	1.86E+01	2.07E-01
F15	avg	1.50E+03	1.51E+03	1.50E+03	1.51E+03	1.57E+03	1.52E+03	1.50E+03	1.52E+03	1.50E+03
F16	min	1.60E+03	1.60E+03	1.60E+03	1.60E+03	1.60E+03	1.60E+03	1.60E+03	1.60E+03	1.60E+03
F16	std	2.78E-01	3.25E-01	3.89E-01	3.18E-01	2.37E-01	1.93E-01	3.43E-01	8.29E-01	4.91E-01
F16	avg	1.60E+03	1.60E+03	1.60E+03	1.60E+03	1.60E+03	1.60E+03	1.60E+03	1.60E+03	1.60E+03
F17	min	1.23E+04	6.15E+03	3.10E+03	4.52E+03	2.83E+03	6.46E+03	2.44E+03	5.98E+03	1.70E+03
F17	std	3.67E+04	1.82E+05	1.08E+05	2.44E+04	8.33E+04	9.05E+04	3.34E+03	1.93E+05	5.73E+01
F17	avg	4.09E+04	9.84E+04	4.01E+04	2.52E+04	3.70E+04	2.11E+05	5.74E+03	4.97E+05	1.78E+03
F18	min	1.93E+03	2.01E+03	5.41E+03	2.01E+03	8.73E+03	1.20E+04	2.40E+03	8.08E+03	1.80E+03
F18	std	3.43E+03	7.75E+03	4.84E+03	9.35E+03	2.25E+03	7.51E+04	1.44E+04	2.89E+03	1.48E+00
F18	avg	5.06E+03	8.60E+03	1.14E+04	1.13E+04	1.25E+04	6.08E+04	2.13E+04	1.25E+04	1.80E+03
F19	min	1.90E+03	1.90E+03	1.90E+03	1.90E+03	1.90E+03	1.90E+03	1.90E+03	1.90E+03	1.90E+03
F19	std	2.92E-01	1.21E+00	9.16E-01	1.13E+00	3.49E+00	2.52E+00	6.18E-01	8.68E-01	3.42E-01
F19	avg	1.90E+03	1.91E+03	1.90E+03	1.90E+03	1.91E+03	1.91E+03	1.90E+03	1.90E+03	1.90E+03
F20	min	2.02E+03	2.15E+03	2.14E+03	2.51E+03	3.46E+03	2.34E+03	2.08E+03	4.41E+03	2.00E+03
F20	std	6.31E+02	5.83E+03	3.02E+03	1.90E+03	2.43E+03	3.52E+03	3.31E+02	8.12E+03	4.96E-01
F20	avg	2.50E+03	1.13E+04	4.46E+03	6.14E+03	7.59E+03	5.95E+03	2.44E+03	1.22E+04	2.00E+03
F21	min	2.30E+03	7.60E+03	3.12E+03	3.09E+03	3.36E+03	1.34E+04	3.24E+03	3.77E+03	2.10E+03
F21	std	2.49E+03	4.00E+05	4.98E+03	8.79E+03	8.35E+03	3.62E+04	3.58E+03	1.58E+05	7.15E+00
F21	avg	4.41E+03	2.68E+05	1.12E+04	1.17E+04	1.27E+04	6.38E+04	7.20E+03	8.62E+04	2.10E+03
F22	min	2.20E+03	2.24E+03	2.22E+03	2.23E+03	2.23E+03	2.24E+03	2.23E+03	2.23E+03	2.20E+03
F22	std	2.50E+00	8.27E+01	6.22E+01	8.31E+01	3.02E+01	7.05E+01	7.24E+01	9.65E+01	9.70E+00
F22	avg	2.20E+03	2.30E+03	2.28E+03	2.32E+03	2.26E+03	2.31E+03	2.30E+03	2.35E+03	2.21E+03
F23	min	2.63E+03	2.50E+03	2.63E+03	2.50E+03	2.50E+03	2.50E+03	2.63E+03	2.50E+03	2.50E+03
F23	std	2.67E-10	4.39E+01	7.24E+00	0.00E+00	6.51E+01	0.00E+00	4.73E-04	2.25E-07	7.88E-02
F23	avg	2.63E+03	2.62E+03	2.64E+03	2.50E+03	2.53E+03	2.50E+03	2.63E+03	2.50E+03	2.50E+03
F24	min	2.52E+03	2.54E+03	2.52E+03	2.53E+03	2.54E+03	2.53E+03	2.52E+03	2.52E+03	2.51E+03
F24	std	2.41E+00	2.71E+01	3.45E+01	2.27E+01	2.50E+01	2.33E+01	2.34E+01	3.43E+01	3.65E+00
F24	avg	2.52E+03	2.57E+03	2.54E+03	2.59E+03	2.58E+03	2.59E+03	2.54E+03	2.56E+03	2.51E+03
F25	min	2.65E+03	2.67E+03	2.66E+03	2.69E+03	2.66E+03	2.70E+03	2.62E+03	2.70E+03	2.61E+03
F25	std	1.64E+01	1.08E+01	1.32E+01	3.05E+00	1.35E+01	0.00E+00	3.73E+01	5.98E-01	7.61E+00
F25	avg	2.67E+03	2.70E+03	2.70E+03	2.70E+03	2.70E+03	2.70E+03	2.66E+03	2.70E+03	2.62E+03
F26	min	2.70E+03	2.70E+03	2.70E+03	2.70E+03	2.70E+03	2.70E+03	2.70E+03	2.70E+03	2.70E+03
F26	std	5.51E-02	1.31E-01	3.16E+01	3.15E+01	6.34E-01	3.43E-01	7.85E-02	6.78E-02	3.80E-02
F26	avg	2.70E+03	2.70E+03	2.71E+03	2.71E+03	2.70E+03	2.70E+03	2.70E+03	2.70E+03	2.70E+03
F27	min	2.71E+03	3.10E+03	2.70E+03	2.71E+03	2.71E+03	2.71E+03	2.70E+03	2.90E+03	2.70E+03
F27	std	1.56E+02	2.39E+01	1.17E+02	8.14E+01	1.21E+02	8.69E+01	1.86E+02	7.09E+01	1.04E+02
F27	avg	2.94E+03	3.12E+03	3.03E+03	2.86E+03	2.76E+03	2.83E+03	2.92E+03	3.07E+03	2.80E+03
F28	min	3.16E+03	3.00E+03	3.17E+03	3.00E+03	3.00E+03	3.00E+03	3.16E+03	3.00E+03	3.00E+03
F28	std	8.07E+00	1.67E+02	5.70E+01	0.00E+00	1.34E+02	0.00E+00	6.01E+01	1.76E+02	5.05E+01
F28	avg	3.18E+03	3.36E+03	3.25E+03	3.00E+03	3.29E+03	3.00E+03	3.21E+03	3.08E+03	3.03E+03
F29	min	3.29E+03	3.37E+03	3.33E+03	3.10E+03	3.79E+03	3.60E+03	3.71E+03	3.10E+03	3.12E+03
F29	std	1.14E+02	1.22E+06	6.77E+05	8.83E+02	2.28E+03	7.42E+02	9.59E+02	1.16E+06	6.47E+05
F29	avg	3.54E+03	5.43E+05	2.18E+05	4.01E+03	5.34E+03	4.55E+03	4.36E+03	3.70E+05	2.08E+05
F30	min	3.49E+03	4.15E+03	3.50E+03	4.47E+03	4.32E+03	4.28E+03	3.82E+03	3.20E+03	3.48E+03
F30	std	3.12E+01	6.30E+02	5.79E+02	8.94E+02	8.99E+02	5.08E+02	6.40E+02	9.08E+02	2.86E+01
F30	avg	3.52E+03	5.02E+03	4.08E+03	5.74E+03	5.09E+03	5.15E+03	4.57E+03	4.79E+03	3.53E+03

**Table 2:** The result of the standard functions of CEC2020 for different algorithms.

		DE	WOA	GWO	HHO	DBO	MBWO	AOO	IVY	LSIVY
F1	min	7.97E+02	1.07E+06	6.99E+03	1.34E+05	5.73E+08	7.20E+08	2.18E+02	1.00E+02	1.00E+02
F1	std	3.03E+03	8.55E+06	1.82E+08	3.17E+05	4.86E+08	1.64E+09	2.87E+03	2.54E+03	2.59E-14
F1	avg	4.53E+03	6.27E+06	8.76E+07	6.57E+05	1.14E+09	3.39E+09	2.61E+03	2.16E+03	1.00E+02
F2	min	1.24E+03	1.38E+03	1.24E+03	1.84E+03	1.48E+03	1.35E+03	1.13E+03	1.80E+03	1.17E+03
F2	std	1.79E+02	4.77E+02	3.74E+02	1.71E+02	2.90E+02	3.07E+02	2.68E+02	2.92E+02	1.29E+02
F2	avg	1.62E+03	2.13E+03	1.70E+03	2.03E+03	2.10E+03	1.97E+03	1.57E+03	2.26E+03	1.29E+03
F3	min	7.17E+02	7.50E+02	7.21E+02	7.65E+02	7.60E+02	7.32E+02	7.18E+02	7.44E+02	7.16E+02
F3	std	4.04E+00	2.36E+01	8.34E+00	1.72E+01	1.49E+01	1.32E+01	7.77E+00	2.79E+01	2.46E+00
F3	avg	7.24E+02	7.84E+02	7.34E+02	7.86E+02	7.80E+02	7.56E+02	7.28E+02	7.86E+02	7.20E+02
F4	min	1.90E+03	1.90E+03	1.90E+03	1.91E+03	1.90E+03	1.91E+03	1.90E+03	1.90E+03	1.90E+03
F4	std	3.33E-01	1.45E+01	9.59E-01	2.13E+00	2.79E+04	4.78E+02	5.21E-01	2.64E+01	2.32E-01
F4	avg	1.90E+03	1.91E+03	1.90E+03	1.91E+03	1.62E+04	2.32E+03	1.90E+03	1.92E+03	1.90E+03
F5	min	1.61E+04	4.60E+03	3.60E+03	4.33E+03	5.32E+03	2.52E+04	2.54E+03	7.15E+04	1.70E+03
F5	std	5.07E+04	1.34E+05	1.67E+05	1.98E+04	2.15E+04	1.07E+05	2.09E+03	1.92E+05	1.02E+02
F5	avg	6.15E+04	1.10E+05	1.11E+05	1.93E+04	2.63E+04	2.32E+05	4.73E+03	4.50E+05	1.81E+03
F6	min	1.60E+03	1.74E+03	1.60E+03	1.63E+03	1.75E+03	1.65E+03	1.60E+03	1.72E+03	1.60E+03
F6	std	6.95E+00	8.46E+01	9.61E+01	1.08E+02	1.08E+02	1.13E+02	8.42E+01	1.41E+02	5.35E+01
F6	avg	1.61E+03	1.85E+03	1.75E+03	1.82E+03	1.90E+03	1.82E+03	1.70E+03	1.87E+03	1.63E+03
F7	min	3.04E+03	5.89E+03	3.54E+03	3.04E+03	2.86E+03	8.36E+03	3.37E+03	3.42E+03	2.10E+03
F7	std	6.00E+02	3.06E+05	5.45E+03	8.24E+03	1.21E+04	8.40E+04	6.12E+03	5.17E+06	4.27E+01
F7	avg	3.67E+03	1.66E+05	1.07E+04	1.14E+04	8.78E+03	9.73E+04	8.19E+03	1.77E+06	2.12E+03
F8	min	2.27E+03	2.24E+03	2.30E+03	2.31E+03	2.39E+03	2.23E+03	2.24E+03	2.30E+03	2.30E+03
F8	std	9.57E+00	3.05E+02	1.89E+01	4.48E+00	5.71E+01	9.29E+01	2.07E+01	5.19E+01	5.46E-01
F8	avg	2.30E+03	2.40E+03	2.32E+03	2.31E+03	2.50E+03	2.39E+03	2.30E+03	2.32E+03	2.30E+03
F9	min	2.67E+03	2.75E+03	2.73E+03	2.61E+03	2.55E+03	2.58E+03	2.74E+03	2.74E+03	2.40E+03
F9	std	2.52E+01	2.29E+01	1.28E+01	7.04E+01	9.18E+01	7.19E+01	5.50E+00	8.99E+00	1.22E+02
F9	avg	2.74E+03	2.78E+03	2.74E+03	2.80E+03	2.67E+03	2.74E+03	2.75E+03	2.75E+03	2.68E+03
F10	min	2.90E+03	2.93E+03	2.90E+03	2.90E+03	2.93E+03	2.95E+03	2.90E+03	2.90E+03	2.90E+03
F10	std	1.97E+01	3.23E+01	1.95E+01	2.33E+01	6.94E+01	6.26E+01	2.39E+01	2.26E+01	2.22E+01
F10	avg	2.92E+03	2.97E+03	2.93E+03	2.93E+03	3.04E+03	3.04E+03	2.93E+03	2.93E+03	2.91E+03

**Table 3:** Performance comparison and simulation time comparison of optimization design for three-bar truss structures using 9 algorithms.

Three-Bar Truss Structure	DE	WOA	GWO	HHO	DBO	MBWO	AOO	IVY	LSIVY
Best	2.64E+02	2.64E+02	2.64E+02	2.64E+02	2.64E+02	2.64E+02	2.64E+02	2.64E+02	2.64E+02
Worst	2.64E+02	2.66E+02	2.64E+02	2.64E+02	2.64E+02	2.65E+02	2.64E+02	2.64E+02	2.64E+02
Std	6.85E-05	8.78E-01	5.98E-03	1.87E-01	1.23E-01	3.66E-01	7.87E-03	8.24E-03	1.89E-09
Mean	2.64E+02	2.65E+02	2.64E+02	2.64E+02	2.64E+02	2.64E+02	2.64E+02	2.64E+02	2.64E+02
Median	2.64E+02	2.64E+02	2.64E+02	2.64E+02	2.64E+02	2.64E+02	2.64E+02	2.64E+02	2.64E+02
Time	4.39E-01	1.79E-01	1.69E-01	4.25E-01	2.38E-01	2.84E-01	2.90E-01	4.10E-01	3.93E-01

**Table 4:** Performance comparison and simulation time comparison of cantilever beam optimization design using 9 algorithms.

Cantilever Beam Design	DE	WOA	GWO	HHO	DBO	MBWO	AOO	IVY	LSIVY
Best	1.34E+00	1.41E+00	1.34E+00	1.34E+00	1.34E+00	1.34E+00	1.34E+00	1.34E+00	1.34E+00
Worst	1.34E+00	1.80E+00	1.34E+00	1.35E+00	1.34E+00	1.35E+00	1.34E+00	1.34E+00	1.34E+00
Std	4.54E-05	1.14E-01	8.80E-05	2.73E-03	1.44E-04	3.64E-03	2.91E-04	5.60E-05	1.97E-06
Mean	1.34E+00	1.53E+00	1.34E+00	1.34E+00	1.34E+00	1.35E+00	1.34E+00	1.34E+00	1.34E+00
Median	1.34E+00	1.51E+00	1.34E+00	1.34E+00	1.34E+00	1.35E+00	1.34E+00	1.34E+00	1.34E+00
Time	2.95E-01	6.76E-02	6.59E-02	1.62E-01	1.32E-01	1.43E-01	1.51E-01	2.89E-01	3.47E-01

**Table 5:** Comparison of pressure vessel design performance and simulation time for 9 algorithms.

Pressure Vessel Design	DE	WOA	GWO	HHO	DBO	MBWO	AOO	IVY	LSIVY
Best	6.07E+03	6.75E+03	6.06E+03	6.43E+03	6.19E+03	6.39E+03	6.06E+03	6.10E+03	6.06E+03
Worst	6.77E+03	1.24E+04	7.34E+03	7.55E+03	7.39E+03	7.48E+03	7.36E+03	7.54E+03	6.41E+03
Std	2.23E+02	1.95E+03	4.36E+02	3.65E+02	3.54E+02	3.75E+02	5.39E+02	4.72E+02	1.34E+02
Mean	6.22E+03	8.84E+03	6.27E+03	6.99E+03	6.39E+03	7.03E+03	6.54E+03	6.76E+03	6.14E+03
Median	6.11E+03	8.25E+03	6.08E+03	6.90E+03	6.29E+03	7.07E+03	6.27E+03	6.77E+03	6.09E+03
Time	4.50E-01	1.76E-01	1.77E-01	4.34E-01	2.47E-01	2.95E-01	2.94E-01	4.69E-01	4.18E-01

## Data Availability

The data supporting the findings of this study are available from the corresponding author upon request. There are no restrictions on data availability.
